# Metabolite-enhanced normothermic machine perfusion improves kidney transplant viability

**DOI:** 10.1172/jci.insight.190185

**Published:** 2025-09-23

**Authors:** Jan Czogalla, Fabian Hausmann, Simon Lagies, Sydney E. Gies, Sabrina Christiansen, Nico Kaiser, Fabian Haas, Yusuke Okabayashi, Dominik Kylies, Smilla Hofmann, Rossana Franzin, Niklas Sabra, Sarah Bouari, Yitian Fang, Gisela Ambagtsheer, Ilka Edenhofer, Silvia Chilla, Anne K. Mühlig, Marina Zimmermann, Milagros N. Wong, Takashi Yokoo, Oliver Kretz, Maja Lindenmeyer, Florian Grahammer, Martin J. Hoogduijn, Ron de Bruin, Malte Kuehl, Sonja Hänzelmann, Bernd Kammerer, Loreto Gesualdo, Stefan Bonn, Robert C. Minnee, Tobias B. Huber, Victor G. Puelles

**Affiliations:** 1 III. Department of Medicine, University Medical Center Hamburg-Eppendorf, Hamburg, Germany.; 2 Hamburg Center for Kidney Health (HCKH), Hamburg, Germany.; 3 Institute of Medical Systems Bioinformatics, Center for Biomedical AI (bAIome), Center for Molecular Neurobiology Hamburg (ZMNH), University Medical Center Hamburg-Eppendorf, Hamburg, Germany.; 4 Core Competence Metabolomics, Hilde-Mangold-Haus, and; 5 Institute of Medical Microbiology and Hygiene, Faculty of Medicine, Medical Center, University of Freiburg, Freiburg, Germany.; 6 Division of Nephrology and Hypertension, Department of Internal Medicine, The Jikei University School of Medicine, Tokyo, Japan.; 7 Section of Nephrology, Department of Emergency and Organ Transplantation, University of Bari, Bari, Italy.; 8 Erasmus MC Transplant Institute, Department of Internal Medicine, Division of Nephrology and Transplantation, Erasmus University Medical Center, Rotterdam, Netherlands.; 9 University Children’s Hospital and University Children’s Research, University Medical Center Hamburg-Eppendorf, Hamburg, Germany.; 10 Department of Clinical Medicine, Aarhus University, Aarhus, Denmark.; 11 Department of Pathology, Aarhus University Hospital, Aarhus, Denmark.; 12 Institute of Organic Chemistry,; 13 BIOSS Centre for Biological Signalling Studies, and; 14 Spemann Graduate School of Biology and Medicine, University of Freiburg, Freiburg, Germany.

**Keywords:** Cell biology, Metabolism, Nephrology, Amino acid metabolism, Apoptosis pathways, Organ transplantation

## Abstract

Normothermic machine perfusion (NMP) has become a valuable tool to expand the pool of transplantable organs. However, the application of NMP to kidneys presents substantial challenges, mostly due to high variability in the composition of currently used perfusion solutions. Here, we provide a multimodal cross-species cellular atlas of kidney injury associated with NMP using a literature-based consensus buffer. This resource provided a systematic framework that was used to develop a metabolite-enhanced perfusion solution, which protected renal proximal tubular cells, improving cellular viability and transplantation outcomes across species, including human kidneys.

## Graphical abstract

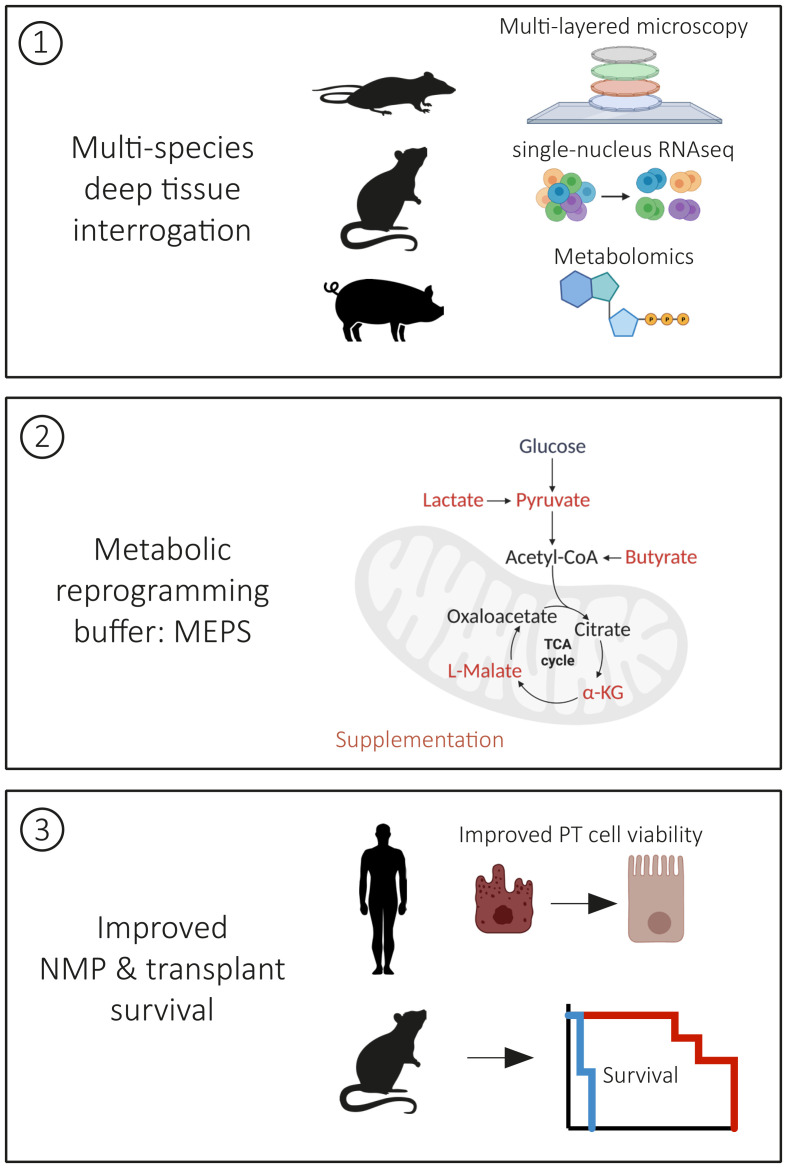

## Introduction

Humanity faces a global shortage of organs for transplantation (1). Implementing strategies that use grafts that would otherwise be discarded can effectively expand the pool of available organs (2). In this context, normothermic machine perfusion (NMP) has demonstrated substantial benefits for liver and lung transplantation, improving organ viability, prolonging storage time, and even reversing cellular damage (2–6). However, this does not extend to all transplantable organs, showing that each organ has a different susceptibility to NMP conditions (i.e., perfusion buffers). For example, clinical trials of NMP for kidney transplantation have confirmed feasibility and safety for 1 hour with comparable results to regular cold storage (7) but have not demonstrated enhancements in storage duration, graft function, tissue viability, or patient survival (8). This indicates that kidney cells may be particularly sensitive to current NMP buffers (9–12). In this context, kidney transplantation provides a unique opportunity to systematically investigate potential shortcomings and propose evidence-based changes. However, most transplant centers have established protocols implemented based on clinical experience, and thereby anecdotal evidence (13). Here, we dissected the origins of cellular injury during kidney NMP performed with a literature-based buffer (hereafter referred to as “consensus buffer”) that represents the majority of current commonly used perfusion solutions. Then, we used a systems biology–based approach that included metabolomic, transcriptomic, spatial proteomic, and histopathologic profiling to develop an atlas of cellular processes triggered by NMP using a consensus buffer across species, including mouse, rat, pig, and human. This resource was leveraged to generate an improved buffer, which we called metabolite-enhanced perfusion solution (MEPS), that mitigated renal tubular epithelial cell injury in vivo, effectively preserving organ integrity and prolonging transplantation viability.

## Results

### Consensus buffer leads to metabolic injury.

**Figure 1:**
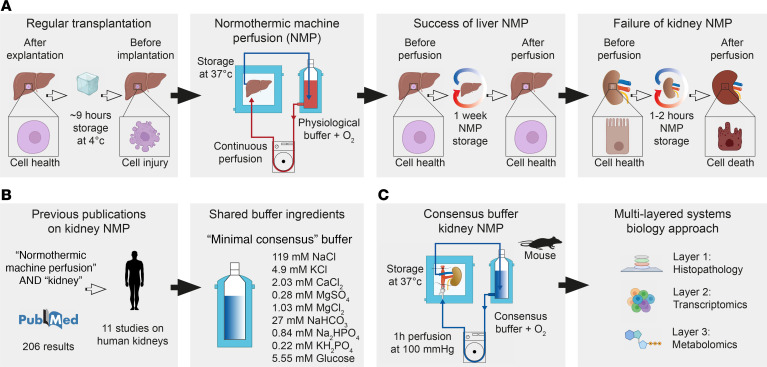
Overview over multilayered analysis of a cross-species approach to understand the shortcomings of kidney NMP. (**A**) Compared with standard organ preservation, liver NMP shows great potential, while kidney NMP does not deliver the same promise. (**B**) A PubMed review of previously published perfusion buffers in human kidney NMP identified shared ingredients and lead to a “minimal consensus” buffer. (**C**) A mouse model of kidney NMP was established using the consensus buffer and resulting tissue analyzed using a multi-layered systems biology approach combining advanced microscopy, single nucleus RNA-Seq (snRNA-Seq) and metabolomics.

The main source of heterogeneity during NMP is the composition of the perfusion solution (13). Compared with liver NMP, kidney NMP shows surprisingly little beneficial effects (Figure 1A). We performed a systematic literature analysis and located the relevant studies on human kidney NMP. We identified a total of 11 studies (7, 8, 10, 14–21), analyzed buffer compositions, identified common ingredients, and defined a baseline consensus-based buffer formulation (Figure 1B), which we used to establish a murine model of kidney NMP toward a systematic dissection of molecular processes (Figure 1C).

**Figure 2:**
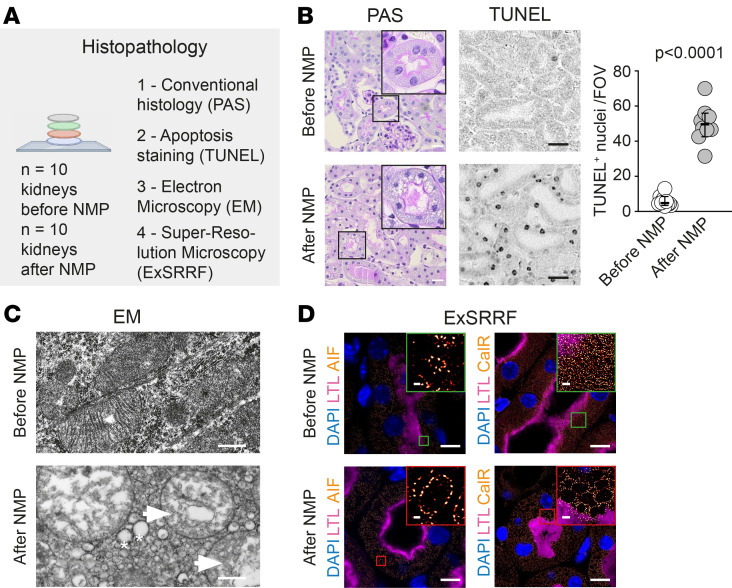
Histopathological injury and increased apoptosis during NMP. (**A**) Histopathology analysis overview. (**B**) Histopathology demonstrated swollen and apoptotic cells in proximal tubules (PTs) after perfusion. Scale bar: 10 μm. TUNEL staining returned significantly increased apoptosis after NMP. Scale bar: 5 μm. Data are shown as a median with 95% CI, using unpaired student’s *t* test. (**C**) Transmission electron microscopy showed damaged mitochondria (arrows) and dilated ER (stars). Scale bar: 500 nm. (**D**) Expansion enhanced super-resolution radial fluctuations (ExSRRF) revealed that swollen and damaged mitochondria and ER were mainly localized in proximal tubules. Scale bar: 5 μm, 1 μm (inserts). LTL, Lotus Tetragonolobus Lectin; AIF, Apoptosis Inducing Factor Mitochondria Associated 1; CalR, Calreticulin.

Next, we used a multilevel histopathological approach to define cellular and organelle injury (Figure 2A). We identified generalized cell swelling and apoptosis (Figure 2B). Ultrastructural analyses using classical transmission electron microscopy and super-resolution microscopy (22, 23) revealed peroxisome induction, as well as endoplasmic reticulum (ER) dilation and mitochondrial swelling (Figure 2C) in association with increased transcriptomic and posttranslational modifications, marking mitochondrial and ER stress (Supplemental Figure 1; supplemental material available online with this article; https://doi.org/10.1172/jci.insight.190185DS1), which were most prominent in the proximal tubules (PTs) (Figure 2D). Together, these findings show that the fundamental consensus composition of the buffers currently used for kidney NMP is insufficient to prevent metabolic stress and cell injury.

### Transcriptional origins of metabolic stress.

**Figure 3:**
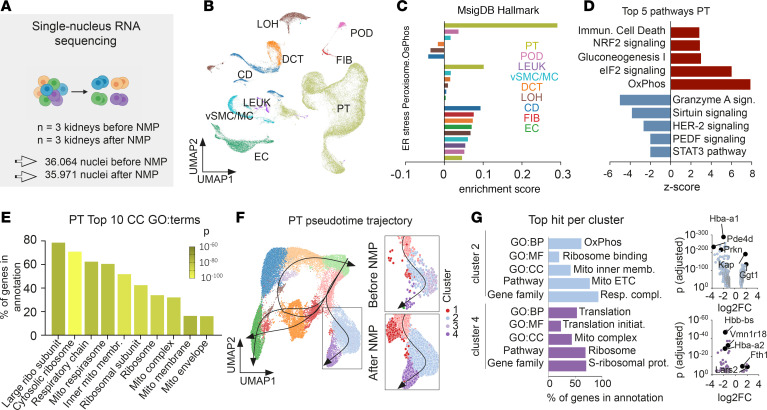
Transcriptomic origins of cellular injury patterns during NMP on single-nuclear level point toward mitochondrial and ER injury in PT subclusters. (**A**) Cell number analyzed using snRNA-Seq of kidneys before and after consensus buffer perfusion. (**B**) Cluster identity. (**C**) An enrichment score of the curated gene sets of the molecular signatures database (MsigDB) applied toward cell types returned PTs as the main affected cell type. (**D**) The top 5 up- and downregulated pathways in PTs show mitochondrial and ER stress. (**E**) Top 10 GO:term analysis of cellular components predicted to be involved in PTs solely returned mitochondrial or ER signals. cc, cellular components. (**F**) Pseudotime analysis in PTs returned 1 lineage significantly altered during NMP with consensus buffer. (**G**) Analysis of the one top signal for 5 different means of analysis (GO:biological process, GO:molecular function, GO:cellular component, pathway and gene family) in subclusters of the affected lineage showed mitochondrial injury in subcluster 2 and ER injury in subcluster 4. Volcano plots of the main individual results for clusters 2 and 4.

To understand the underlying cell type–specific transcriptomic mechanisms of injury, we performed single-nucleus RNA-Seq (snRNA-Seq; *n* = 72,035 cells from *n* = 6 mice) (Figure 3A). Nine main cell types were identified and used for differential gene transcription within the individual clusters (Figure 3B and Supplemental Figure 2). Next, the Human Molecular Signatures Database (24) was used to select gene sets associated with the main structural changes at a single-cell level (i.e., oxidative phosphorylation [OxPhos], peroxisome, and the unfolded protein response), confirming that PTs represent the primary injured cell type (Figure 3C). Furthermore, we observed an enrichment in cytokine and nucleotide transcription signaling (Supplemental Figure 3), both of which have been associated with increased susceptibility to kidney graft rejection (25–27).

Next, we focused on the top 5 regulated pathways in PTs and discovered an upregulation of OxPhos and eukaryotic Initiation Factor 2 (eIF2) signaling, along with the downregulation of Granzyme A and Sirtuin signaling (Figure 3D). These findings support patterns of mitochondrial injury and ER stress in PTs at a transcriptional level (Figure 3E). In addition, we identified multiple dynamic cell states associated with NMP using consensus buffer (Figure 3F and Supplemental Figure 3), showcasing a biphasic process that is first triggered by mitochondrial injury and followed by ER stress (Figure 3G). Together, our data reveal a profound transcriptional disruption of metabolic processes in PTs after NMP with consensus buffer.

### Metabolite deficiencies induce cell injury.

**Figure 4:**
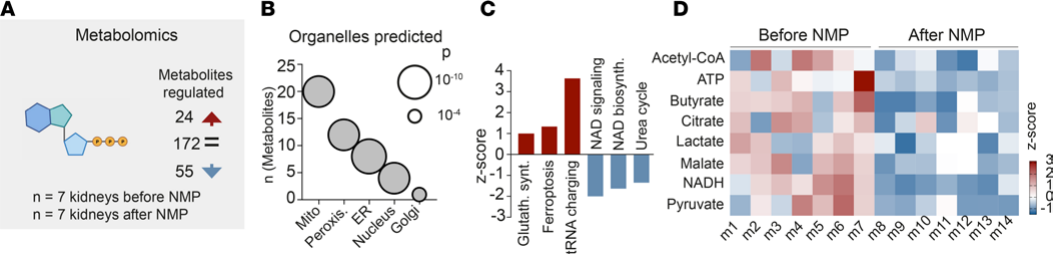
Loss of citric acid cycle metabolites leads to cellular injury during NMP. (**A**) Metabolomics of tissue before vs. after NMP with consensus buffer showed 24 significantly upregulated and 55 significantly downregulated metabolites. (**B**) Metabolite organelle mapping identified mitochondria, peroxisomes and ER as the 3 top organelles predicted to be affected by NMP with consensus buffer. (**C**) Pathway analysis on metabolomics results returned mainly downregulation of citric acid cycle products. (**D**) Metabolites from the citric acid cycle showed global downregulation by perfusion. Multiple unpaired *t* tests were used for statistical analysis.

As structural and transcriptional changes suggested metabolic stress as a common injury path, we performed unsupervised metabolomic profiling, which detected 24 upregulated and 55 downregulated metabolites during NMP using consensus buffer (Figure 4A). These data predict subcellular injury targets (Figure 4B) and altered cellular processes, namely upregulation of transfer RNA (tRNA) charging and ferroptosis, and downregulation of nicotinamide-adenine-dinucleotide (NAD) signaling and NAD biosynthesis (Figure 4C). This metabolite deficiency was directly represented in the citric acid cycle (Figure 4D). We did not detect a change in citric acid cycle component enrichment in the fluids collected during perfusion, suggesting increased tissue metabolic dysregulation rather than excretion into the urine or the buffer (Supplemental Figure 4).

Together, these findings provide a mechanistic explanation to structural and transcriptomic alterations after NMP with consensus buffer, featuring PTs as key contributors to organ dysfunction, and identifying mitochondria as the primary target (28–31). Furthermore, this completes our murine atlas for kidney injury after NMP with consensus buffer, providing a valuable resource to systematically propose modifications that may improve buffer performance.

### Conserved injury patterns across species.

To increase translational value, we conducted a cross-species validation comparing rat and pig kidneys perfused with consensus buffer under the same condition as in mice. The patterns of renal cell injury in the rat (Supplemental Figure 5) and pig (Supplemental Figure 6) confirmed what we observed in mice. Briefly, ultrastructural analysis revealed swollen mitochondria in rat PTs. We also observed a high level of transcriptional congruence in altered molecular pathways in rat and pig PTs compared with mice. Remarkably, eIF2 signaling, ferroptosis, and Sirtuin signaling were consistently regulated in the same direction across all 3 species.

Metabolomic profiling further reinforced our findings, showing substantial overlap between species in pathways such as glutathione synthesis, tRNA charging, NAD signaling, and urea cycle signaling, as well as affected organelles (e.g., mitochondria, peroxisomes, and ER). Notably, interspecies integration of snRNA-Seq and metabolomics data reveal a remarkable overlap of 243 pathways (of 515 total; 47%) and 265 pathways (of 320 total; 82%) in the context of comparable physiologic parameters during perfusion (Supplemental Figure 7). Together, our data demonstrate a high level of interspecies overlap in functional and cellular responses after NMP with consensus buffer.

### MEPS.

**Figure 5:**
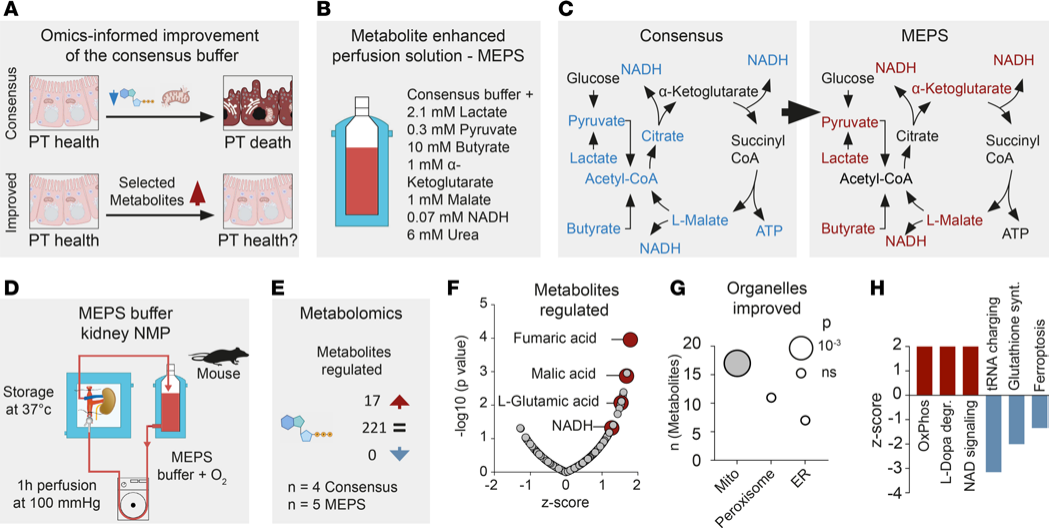
Metabolic reprogramming with citric acid cycle metabolites improves kidney viability after NMP. (**A**) We hypothesized that replenishment of the citric acid cycle intermediates lost during NMP would lead to improved mitochondrial health, leading to better tissue integrity. (**B**) We named the buffer containing these additions MEPS. (**C**) Mapping of downregulated metabolites onto the citric acid cycle showed both input- and output of the cycle were downregulated. Therefore, we replenished the main input and output metabolites of the citric acid cycle to generate MEPS. (**D**) MEPS was tested during mouse NMP. (**E**) Metabolomics of kidneys after perfusion with MEPS showed 17 significantly upregulated metabolites. Multiple unpaired *t* test was used for analysis of significance. (**F**) Main upregulated metabolites were closely related to mitochondrial processes. (**G**) Metabolite organelle mapping returned mitochondria as the sole organelle predicted to be improved by MEPS. Multiple unpaired *t* tests were used for statistical analysis. (**H**) Pathway analysis showed partial reversal of deleterious changes seen with consensus buffer.

Next, our consensus buffer was reengineered supplementing metabolites to compensate for the deficiencies detected in the citric acid cycle (Figure 5, A–C). This process led to the development of a MEPS. First, we tested MEPS in an ex-vivo kidney slice model, which provided a rapid and reductionist approach to assess the effect of different buffer formulations on renal tissues (32). We observed global decreases in mitochondrial injury markers (i.e., IDH1), ER stress (i.e., ATF4), apoptosis (i.e., CHOP), and necroptosis (i.e., RIPK3) (Supplemental Figure 8, A and B).

Then, the effects of MEPS and consensus buffer were compared in mice after NMP (Figure 5D). We identified 17 metabolites that were upregulated and no downregulated metabolites (Figure 5E), confirming that our supplementation strategy was successful. Notably, the upregulated signals predominantly consisted of constituents or products associated with the citric acid cycle (Figure 5F). As expected, in silico organelle prediction based on regulated metabolites identified mitochondria as the most affected organelle (Figure 5G). Furthermore, MEPS mitigated the adverse effects of the consensus buffer, specifically counteracting the overactivation of tRNA charging and ferroptosis, while effectively increasing NAD signaling (Figure 5H). Quantitative PCR (qPCR) analysis of perfused tissues demonstrated reduced expression of mitochondrial, ER, apoptosis, and necroptosis mRNA signals with a corresponding improvement in posttranslational mitochondrial and ER stress markers (e.g., reduced ERK and eIF2α phosphorylation) and preserved cellular and ultrastructural integrity (Supplemental Figure 8, C–F). It is worth noting that MEPS induced mild increases in vascular resistance during perfusion while urine volume remained unchanged (Supplemental Figure 8, G–I), suggesting minimal impact on perfusion conditions. Together, our data show that MEPS substantially enhanced kidney structural and transcriptional integrity during NMP.

### MEPS prevents protein dysregulation.

**Figure 6:**
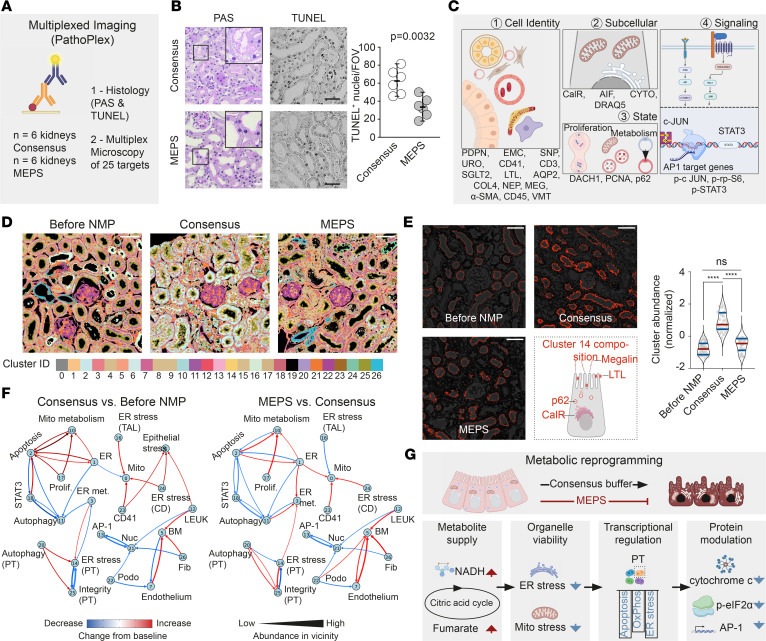
Pathology-oriented multiplexing reveals mechanisms of metabolic reprogramming via MEPS. (**A**) Tissue analysis after MEPS perfusion was performed using histopathology and pathology-oriented multiplexed imaging. (**B**) Histopathology showed partial reversal of deleterious effects seen with consensus buffer. Scale bar: 10 μm. TUNEL staining revealed significantly reduced apoptosis after MEPS perfusion. Scale bar: 30 μm. Data are shown as median with 95% CI. Unpaired student’s *t* test was used for statistical analysis. (**C**) Overview over the targets for multiplexed imaging. (**D**) Pixel-based cluster analysis identified 27 individual clusters. Scale bar: 50 μm. (**E**) Cluster 14 was defined by its top contributors, including Megalin, p62 (ubiquitin-binding protein 62), CalR, and LTL and provides a clear example of cellular processes preserved by MEPS. Data are presented as median with 95% CI. Scale bar: 50 μm. Brown-Forsythe and Welch ANOVA were used for statistical analysis. (**F**) Using the clusters, immediate proximity analysis predicted deleterious mitochondrial signals to negatively affect metabolism in PTs after NMP with consensus buffer but not with MEPS. (**G**) In summary, MEPS perfusion improved metabolite supply, reduced ER-and mitochondrial stress, and led to transcriptional regulation and spatial protein modulation, which in turn resulted in preserved organ viability. AP-1, Activating protein-1; BM, Basement membrane; CD, Collecting duct; CD41, Cluster of differentiation 41; ER met, Metabolism of the ER; Fib, Fibroblast; Leuk, Leucocyte; Mito, Mitochondria; Nuc, Nucleus; Podo, Podocyte; Prolif, Proliferation; PT, Proximal tubule; STAT3, Signal transducer and activator of transcription 3; TAL, Thick ascending limb of the Loop of Henle.

As a validation strategy, we used histopathology and pathology-oriented multiplexing (PathoPlex) (33) to investigate the expression and regulation of 25 protein targets selected to determine cell identity, subcellular organelles, cell state, and pathway activation in the same specimen (Figure 6, A–C, and Supplemental Table 2). Histopathology showed significant improvement with MEPS (Figure 6B). Unsupervised pixel-based analysis generated 27 different biologically relevant clusters (Supplemental Table 3). MEPS successfully prevented the development of pathogenic cluster signatures induced by NMP with consensus buffer (Figure 6D). For example, cluster 14 (C14) featured PT and ER stress–specific signals as top contributors and showed a significant increase in abundance during NMP with consensus buffer but remained unchanged with MEPS (Figure 6E). Cluster proximity was used to generate an interaction network, confirming that MEPS perfusion increased mitochondrial metabolism, enhanced mitochondrial signaling toward the ER, and reduced cell death; this provided an independent validation of metabolomic and transcriptomic changes at the protein level at subcellular resolution (Figure 6F). Thus, these data confirm that MEPS preserves ER and mitochondrial integrity (Figure 6G), suggesting that MEPS may increase graft viability.

### MEPS improves transplantation in vivo.

**Figure 7:**
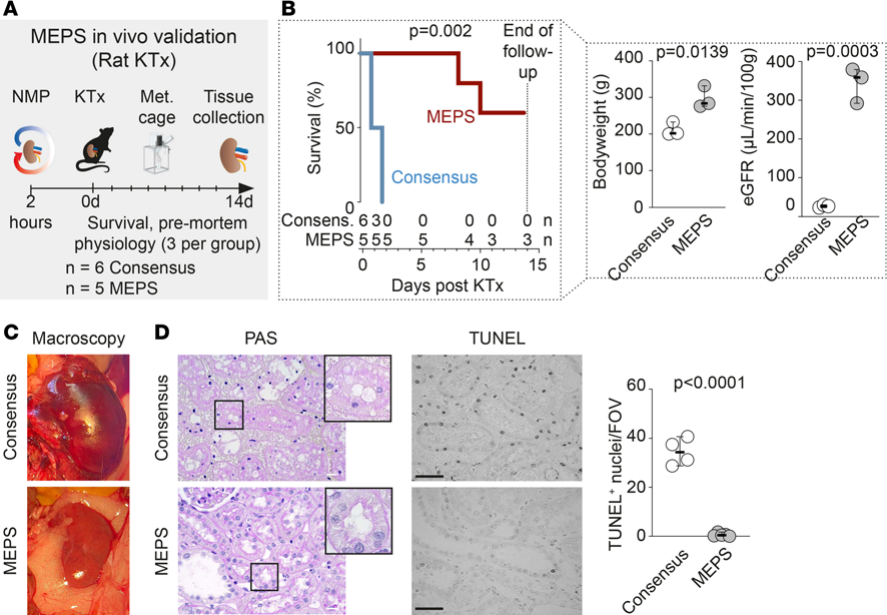
MEPS improves survival in a rat model of kidney transplantation after NMP (**A**) In a rat model of NMP followed by transplantation, MEPS was compared with consensus buffer and animals followed for 14 days after surgery. (**B**) Survival of animals was significantly improved after MEPS perfusion. One day before death or sacrifice, bodyweight was significantly higher in MEPS perfused animals. In addition, estimated glomerular filtration rate (eGFR) was significantly improved after MEPS. Data are shown as median with 95% CI. Log-rank test and unpaired student’s *t* test has been used for statistical analysis. (**C**) There was an evident macroscopic improvement in the kidneys at time of sacrifice with MEPS compared with consensus buffer after NMP. (**D**) Histopathology showed reduced tissue injury after MEPS. Scale bar: 20 μm. TUNEL staining returned significantly less apoptotic nuclei with MEPS compared with consensus buffer after NMP. Scale bar: 30 μm. Data are shown as median with 95% CI, unpaired student’s *t* test has been used for statistics.

Next, we performed kidney transplantation in rats after NMP using either consensus buffer or MEPS (34). Rat kidneys were perfused for 2 hours before transplantation, which would be equivalent to about 54 hours in humans (35). Animals were closely monitored for 14 days. If signs of distress were identified, animals were removed from experiments as per legal protocol. The maximal follow-up time of our approved protocol was 14 days, after which all animals were sacrificed regardless of their overall health status (Figure 7A).

Rats that received kidneys perfused with MEPS showed a substantially higher survival rate during the experimental period compared with those that received kidneys perfused with consensus buffer, reflecting preserved bodyweight and renal function (Figure 7B). Urinalysis performed 1 day prior to death or sacrifice demonstrated a decrease in casts, WBCs, erythrocytes, and protein in urine following MEPS compared with consensus buffer (Supplemental Figure 9, A and B). At the time of sacrifice, MEPS-perfused kidneys largely resembled a healthy organ, but consensus-perfused kidneys appeared swollen and inflamed (Figure 7C). Importantly, we observed a significant prevention of apoptosis in MEPS-perfused kidneys compared with consensus buffer (Figure 7D). We identified healthier mitochondria in PTs and enhanced cellular survival in MEPS-perfused kidneys compared with consensus buffer (Supplemental Figure 9, C and D). It is worth noting that the cause of premature death of the 2 animals in the MEPS group was postrenal failure and thus most likely surgical error (Supplemental Figure 9E). Together, our data suggest that MEPS enhances cellular and organ viability, improving survival after transplantation.

### MEPS preserves pig and human kidneys.

**Figure 8:**
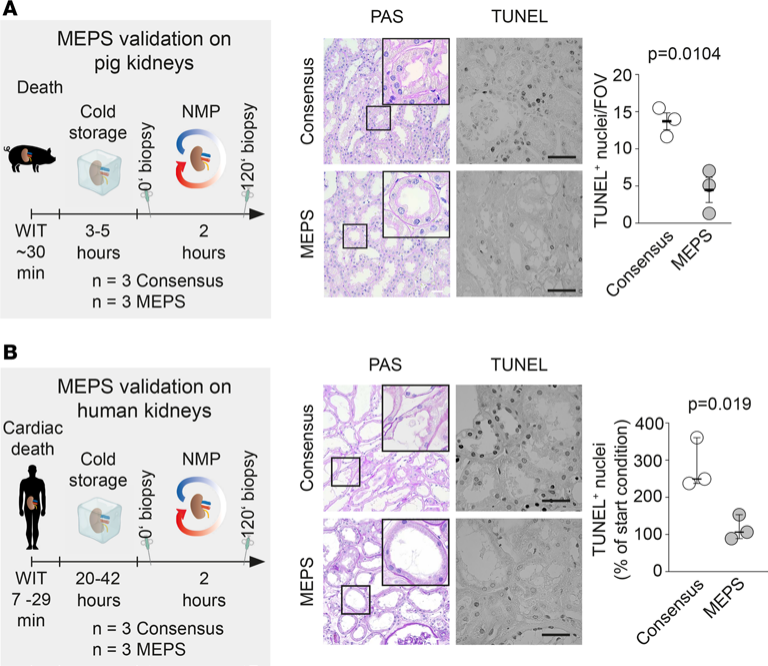
MEPS improves kidney cellular viability in pigs and humans. (**A**) Pig kidneys were stored at 4°C for 3–5 hours and were afterward perfused, comparing MEPS to consensus buffer. Histopathology demonstrated improved cellular structures. Scale bar: 20 μm. TUNEL staining showed significantly less apoptotic nuclei. Scale bar: 30 μm. Data are shown as median with 95% CI, using unpaired student’s *t* test for statistical analysis. (**B**) Human kidneys discarded for regular transplantation were stored at 4°C for up to 42 hours and then randomized to either MEPS or consensus buffer NMP. Histopathology showed improved tissue viability after MEPS. Scale bar: 20 μm. TUNEL staining confirmed significantly less apoptotic nuclei after MEPS. Scale bar: 30 μm. Data are shown as median with 95% CI, using unpaired student’s *t* test for statistical analysis.

To extend our findings to larger organs, we evaluated MEPS in pig kidneys after NMP. Experimental organs were delivered from the abattoir and subjected to 30 minutes warm and 3–5 hours cold ischemia to mimic a clinical scenario (Figure 8A). Structural integrity and apoptosis were used as surrogate markers of organ viability. While MEPS preserved cellular structure and prevented apoptosis in PTs, consensus buffer led to swollen, damaged, and apoptotic PTs (Figure 8A).

To evaluate the potential clinical translation of our findings, we assessed MEPS during NMP of discarded human kidneys. Following donation after cardiac death, 6 kidneys were stored at 4°C for a maximum of 42 hours and subsequently subjected to NMP for 2 hours, with randomization into 2 groups: consensus buffer or MEPS (Figure 8B). Among the donors, sex, creatinine levels, urine output, and kidney weight were similar before randomization (Supplemental Figure 10, A–C). Prior to NMP, kidneys already exhibited varying degrees of tissue damage that may be explained by warm and cold ischemic times (average, 15.0 minutes and 28.87 hours, respectively) but was not statistically significant between experimental groups (Supplemental Figure 10D).

While MEPS-perfused kidneys showed improved tissue integrity and stable levels of apoptosis, consensus-perfused kidneys presented increases in apoptosis and marked deterioration of cellular integrity (Figure 8B). During NMP, we observed a nonsignificant trend toward increased vascular resistance with MEPS (Supplemental Figure 10E). This final experiment confirms the clinical potential of MEPS to extend organ viability and even suggests a potential for organ regeneration.

## Discussion

NMP is revolutionizing the field of organ transplantation, promising extended storage times, enhanced functional testing and practical ex vivo pharmacological interventions, thereby expanding the number of successful transplantations. However, its application to kidneys, the most frequently transplanted organ (1), has thus far yielded unsatisfactory outcomes. To address this, we generated a subcellular multimodal atlas of kidney NMP that defined citric acid cycle metabolite deficiencies as the main injury trigger, providing a mechanistic explanation for the rapid kidney deterioration ex vivo. This resource was used to generate MEPS, which improved graft viability across various species (including humans) and improved posttransplantation survival in rats.

The clinical state-of-the-art in the field of kidney NMP is the use of perfusion solutions based on history, pathophysiological rationale, and clinical experience. This has resulted in marked heterogeneity of the composition in clinical practice (13). Our study proposes a conceptual change that starts with a systematic, reproducible, and accessible framework derived from a multispecies cell atlas of kidney NMP, which can be leveraged to provide evidence-based recommendations to optimize buffer composition and in this study: the generation of MEPS. While our study has limited sample sizes in the experiments conducted in pig and human kidneys, we believe our findings are confirmed by the large consistency of the results across species (i.e., mouse and rat).

We believe that the systems-based approach described in this manuscript may serve as a valuable model for the design of future studies in other organs. To begin with, many current studies rely on a single animal species. Integrating findings across multiple species — from mouse and rat to pig and human — within the same study, wherever feasible, will substantially enhance the translatability of results. Moreover, systematically deconstructing perfusion buffers, assessing the relevance of individual components, and subsequently reassembling them will be a promising strategy to optimize organ-specific perfusion protocols. Finally, the combined analysis of transcriptomic, metabolomic, and proteomic data through integrated multi-omics approaches holds great potential for uncovering novel mechanistic insights.

Recent work has underlined the importance of inter-organ crosstalk for human biology and its role in maintaining a healthy metabolism. During NMP, organs are perfused ex vivo isolated from other organs and thus depend upon the exogenous delivery of the correct signals for cell homeostasis and survival. Our results emphasize the importance of liver and gut metabolites (i.e., butyrate, lactate) for the viability of kidneys and especially PTs (36–38). Given the technical similarities during perfusion, it is likely that our approach will be relevant to other transplant organs, including liver, heart, and lung.

Graft rejection and inflammation are critical aspects of allotransplantation. The experimental approach employed in this study — including perfusion and tissue analysis without subsequent transplantation (in mouse, pig, and human models) as well as syngeneic transplantation (in rats) — does not allow assessment of potential MEPS-related effects on transplant immunity. Future studies will be required to address this important question.

In conclusion, this study identified the molecular origins of metabolic cell injury during kidney NMP, providing a framework to reengineer perfusion solutions, thereby improving organ viability. While we utilize the kidney as an example, this approach can serve as a blueprint for future studies in other organs.

## Methods

### Sex as a biological variable

In mouse and rat studies, only male animals were used, as the surgical method is hampered by the female uterus bicornis in rodents. In pig (sex unknown in abattoir kidneys) and human (sex given in Supplemental Figure 10, 33% female) studies, we took care to exclude sex as a biological variable.

### Procedures

#### Buffer composition.

For all procedures, find buffer compositions in Supplemental Table 1.

#### Mouse kidney perfusion.

Perfusion was performed in the same manner and on the same equipment as previously described (32, 39). The kidney was connected to a pressure-controlled perfusion circuit and perfusion was carried out for 1 hour (h) at 100 mmHg. The perfusate was kept at 37°C and continuously enriched via dialysis with 95% O_2_ and 5% CO_2_. For further surgical and technical details, please refer to the previous publications (32, 39). After perfusion, tissue was either snap-frozen in liquid nitrogen, placed in 4% PFA for diffusion-fixation, or RNAlater (AM7021, Qiagen) for mRNA analysis.

#### Mouse kidney slice incubation.

The procedure was performed as previously described (32). Briefly, decapsulated mouse kidneys were cut into 1 mm–sized slices with a razor blade–based tissue chopper and incubated in 3D-printed slice incubation chambers for 30 min at 33°C in buffer continuously enriched with carbogen, a mixture of 95% O2 and 5% CO_2_. After incubation, tissue was placed in RNAlater (AM7021, Qiagen) for mRNA analysis.

#### Rat kidney perfusion and transplantation.

Perfusion and syngeneic transplantation were carried out as previously described (34). Briefly, kidneys were connected to a pressure-controlled perfusion circuit and perfusion was carried out for 2 h at 100 mmHg. The perfusate was kept at 37°C and continuously enriched via dialysis with 95% O_2_ and 5% CO_2_. After perfusion, kidneys were implanted as previously described (34). Rats were kept under standard conditions as described above with free access to food and water. Animals were weighed and checked 2 times daily following a 13-item score sheet (score, 0–19) (https://doi: 10.1111/aor.13799) for the fulfillment of animal ethics and survival under pain- and stress-free conditions. Animals that did not fulfill the criteria of the score sheet and reach a score of at least 7 were discussed with veterinarians and animal caretakers for the option of potential medical treatment or potential for resolution of the medical condition. If this was not seen in mutual agreement, animals were removed from the experiment. The follow-up period after surgery was 14 days, after which animals were anesthetized and kidneys explanted. The experiment was started with *n* = 6 rats in the consensus group and *n* = 5 animals in the MEPS group. At 24 hours after surgery, only 3 animals in the consensus group were alive, and urine, bodyweight and eGFR measurements could be taken ~24 h before exitus. In the MEPS group, 3 animals survived the whole time of follow up and had urinalysis, body weight, and eGFR measured 24 h before exitus. For *n* = 4 consensus and *n* = 5 MEPS animals, kidney tissue was taken after exitus. Two consensus animals were excluded from this analysis, as they were found dead after multiple hours and rigor mortis had already set in, rendering the value of tissue analysis low. After necropsy, kidney tissue of the remaining animals was split and parts further processed for FACS L/D staining, snap-frozen in liquid nitrogen and placed in 4% PFA for diffusion fixation.

#### Pig kidney perfusion.

Perfusion was performed as previously described (40). Briefly, kidneys were received after slaughter for a warm ischemia time of ~30 minutes and immediately flushed with 500 mL 1 × PBS with 2 mL Heparin 10,000 IE/mL added. Kidneys were stored at + 4°C for 3–5 h, after which normothermic perfusion was initiated. Perfusion took place for 2 h at 100 mmHg at 37°C. The perfusate was continuously enriched with 95% O_2_ and 5% CO_2_. Biopsies were taken at the beginning and end of perfusion. Biopsy tissue was either snap-frozen in liquid nitrogen or placed in 4% PFA for diffusion fixation.

#### Human kidney perfusion.

Perfusion was performed similarly as previously described (18, 41). Briefly, the NMP system consists of a centrifugal pump head (BPX-80 Bio-Pump Plus, Medtronic, Minneapolis, MN, USA) equipped with a pump drive (BVP-BP, Harvard Apparatus, Germany), an oxygenator with a heat exchanger (Hilite 1000, Medos, Germany), and a thermocirculator (E100, Lauda, Germany). A flow probe (73-4755, Harvard Apparatus, Germany) is connected in-line with the kidney and a pressure sensor (APT300, Harvard Apparatus, Germany) is directly connected to the renal artery cannula (Organ Recovery Systems, Itasca, USA). The setup is controlled by a commercially available electronic controller (PLUGSYS Servo Controller for Perfusion, Harvard Apparatus, Germany) which enables both flow and pressure-directed perfusions. The kidneys were perfused with the different buffers at 37°C with a controlled pressure of 70 mmHg. During NMP, carbogen (95% O_2_ and 5% CO_2_) was supplied via the oxygenator at a flow rate of 500 mL/min.

### Histopathology

#### Periodic acid–Schiff staining.

Sections (2 μm thick) were cut from tissue samples embedded in paraffin. Staining was performed with periodic acid–Schiff reagent (Incubation times: 1 hour in Schiff’s reagent and 3 min hematoxylin). Microscopy was carried out using a THUNDER 3D Live Cell and 3D Cell Culture system (Leica Microsystems).

#### TUNEL staining.

Sections (2 μm thick) were cut from tissue samples embedded in paraffin. The Deadend Colorimetric TUNEL System (G7130, Promega) was used according to the manufacturer’s specifications on paraffin-embedded tissue samples. The incubation time of proteinase K was 20 min, and the staining time of DAB was 12 min. TUNEL^+^ nuclei were counted in a blinded manner. For each condition, 15-20x microscopic images per sample were evaluated. Microscopy was carried out using a THUNDER 3D Live Cell and 3D Cell Culture system (Leica Microsystems).

#### Transmission electron microscopy.

Formalin-fixed tissue was dissected and cut into pieces of 1 mm^3^, fixed briefly at room temperature and for 48 h at 4°C in a buffer containing 4.0% PFA, 2.5% glutaraldehyde, and 0.1M cacodylate (pH 7.4). The tissue blocks were contrasted using 1% OsO_4_ (Roth 7436.1) at room temperature for 1 h and 1% uranyl acetate (Polysciences 21447-25) in 70% ethanol at room temperature for 1 h. After dehydration, tissue blocks were embedded in epoxy resin (Durcopan ACM, Sigma-Aldrich 44611), and ultrathin sections of 50 nm thickness were cut using a Leica EM UC6 ultramicrotome (Leica Microsystems). The sections were imaged using a Zeiss 910 transmission electron microscope and analyzed using ITEM software Version 5.2 (build 4768).

#### Immunofluorescence microscopy.

For immunofluorescence microscopy, 2 μm–thick sections were sectioned from tissue samples embedded in paraffin. The sections were blocked in PBS containing 5% BSA and incubated for 45 min with primary antibodies at room temperature as indicated below. Fluorophore-conjugated secondary antibodies (Thermo Scientific, Germany) were applied for 30 min. Images were acquired using a Zeiss ApoTome (Zeiss, Oberkochen, Germany). Primary antibodies used were: Nephrin (Progen, GP-N2), LTL (Vector Laboratories, B-1325-2), pS51-eIF2a (Cell Signaling, 9721). The secondary antibodies were used at a dilution of 1:200 to 1:300. Antibodies: anti-guinea pig IgG Alexa Fluor 488 (Thermo Fisher Scientific; A11073), anti-mouse IgG Alexa Fluor 647 (Thermo Fisher Scientific; A31571), and anti-rabbit IgG Alexa Fluor 555 (Thermo Fisher Scientific; A31572).

#### Expansion-enhanced super-resolution radial fluctuations.

The methodology of expansion-enhanced super-resolution radial fluctuations (ExSRRF) has previously been described (22, 23, 42). Briefly, immunolabeled sections first underwent anchoring treatment (0.1 mg/mL Acryloyl-X (Invitrogen A20770) overnight at room temperature. Next, sections were embedded into a gelling solution (1× PBS, 2 M NaCl, 8.625% sodium acrylate (Sigma-Aldrich 408220), 2.500% acrylamide (Sigma-Aldrich A3553), 0.100% N-N′-methylenbis-(acrylamide) (Sigma-Aldrich 146072), 0.010% 4-hydroxy-2,2,6,6-tetramethyl-piperidin-1-oxyl (Sigma-Aldrich 176141), 0.200% N,N,N′,N′-tetramethylethylenediamine (Sigma-Aldrich T9281), and 0.200% ammonium persulfate (PanReac AppliChem, A1142). Embedded sections in the gelling solution were then incubated at 4°C for 30 min, after which a gelling chamber was constructed, consisting of 2 coverslips as spacers and a third coverslip on top of the tissue. Then, sections were incubated in a humidified oven at 37°C for 2 h to complete gelation. After this, the gelling chambers were removed, and specimens were incubated in 8 U/mL proteinase K (Sigma-Aldrich P2308) in a Tris/EDTA-based digestion buffer (50 mM Tris (pH 8), 25 mM EDTA, 0.5% Triton X-100 and 0.8 M NaCl) at 60°C for 4 h. After completing the digestion step, gel-embedded tissue sections were placed in double-deionized water for 60 min for isotropic expansion. The tissues were then mounted in glass-bottom chamber slides (Ibidi μ-Slide 2-well glass bottom; catalog 80287) for subsequent imaging. Post-expansion imaging of tissues was performed using the THUNDER Imager 3D Live Cell and 3D Cell Culture (Leica Microsystems) in combination with a ×63 objective (NA, 1.10) after optimizing the LED intensity and exposure times. To enable post-expansion SRRF processing, time stacks (each consisting of 50 individual frames per final ExSRRF-image) were obtained for each ROI.

File navigation and processing including histogram and LUT-adjustments as well as SRRF processing of raw data was performed using the Fiji imaging software Version 2.3.0/1.53q (Max Planck Institute of Molecular Cell Biology and Genetics) in combination with the NanoJ-SRRF plug-in. Raw time-stacked files were processed using the following settings: Ring radius, 0.5–2.0; radiality magnification, 3–10 (depending on the desired magnification); axes in ring, 2–8 (depending on the desired magnification); temporal analysis; temporal radiality, average radiality; remove positivity constraint ‘disabled’; renormalize ‘disabled’; do gradient smoothing ‘active’; weighting, do intensity weighting ‘active’; do gradient weighting ‘disabled’; corrections, minimize SRRF patterning ‘active’; fast linearize SRRF ‘disabled’.

### PathoPlex

Multiplexed imaging was performed as recently reported (33). A total of 25 targeted primary antibodies were used in this study as detailed in Supplemental Table 2. The secondary antibodies were used at dilution of 1:200 to 1:300. Antibodies included: anti-guinea pig IgG Alexa Fluor 488 (Thermo Fisher Scientific; A11073), anti-guinea pig IgG Alexa Fluor 555 (Thermo Fisher Scientific; A21435), anti-mouse IgG Alexa Fluor 488 (Thermo Fisher Scientific; A21202), anti-mouse IgG Alexa Fluor 555 (Thermo Fisher Scientific; A31570), anti-mouse IgG Alexa Fluor 647 (Thermo Fisher Scientific; A31571), anti-rabbit IgG Alexa Fluor 488 (Thermo Fisher Scientific; A21206), anti-rabbit IgG Alexa Fluor 555 (Thermo Fisher Scientific; A31572), anti-goat IgG Alexa Fluor 488 (Thermo Fisher Scientific; A11055), anti-goat IgG Alexa Fluor 555 (Thermo Fisher Scientific; A21432), anti-rat IgG Alexa Fluor 488 (Thermo Fisher Scientific; A21208), streptavidin Alexa Fluor 488 (Thermo Fisher Scientific; S11223), and streptavidin Alexa Fluor 555 (Thermo Fisher Scientific; S21381).

Computational analysis of the multiplex data was performed using the *spatiomic* Python package (33), which uses pixel-based dimensionality reduction and unsupervised clustering to identify biologically meaningful protein co-expression patterns. The full documentation for the spatiomic analysis library can be found at https://spatiomic.org/

The precise alignment of multicycle fluorescence microscopy images is essential for accurate pixel-based image analysis. To achieve this, we employed an iterative registration framework called Elastix (43), complemented by a Python wrapper package named Pyelastix (accessible at https://github.com/almarklein/pyelastix). The transformation parameters were computed based on the nuclear channel obtained in each cycle, where the initial cycle served as the reference to which all subsequent cycles were aligned. All nuclear channels were normalized within a range of 0–1 and normalized correlation was used as the optimization metric. The registration process involved iterative steps at 4 different resolution levels, each comprising 250 iterations. To account for x and y offsets, as well as rotation, the rigid Euler transform was utilized. Following the computation of transformation parameters, the channels’ stacks for all image positions were cropped to the regions where all cycles overlapped.

#### Data preprocessing.

We analyzed 4 kidneys per condition with 8 imaging positions, representing 3 groups: “Before NMP,” “Consensus,” and “MEPS.” This resulted in 96 multiplexed image stacks of 25 channels, providing comprehensive data for analysis. To ensure computational efficiency without sacrificing data representation, we created a subsample by selecting 5% of pixel positions. We verified that the marker distribution within this subset closely resembled that of the full dataset.

To improve the dynamic range of the images and eliminate outlier pixel intensities, we applied channel-wise clipping, setting the minimum percentile to 5% and the maximum percentile to 99.9%. We then performed z-scoring for each marker channel to account for different means and standard deviations and to transform pixel intensities to a comparable scale. To facilitate further data processing and improve interpretability, we performed channel-wise normalization, scaling values between 0 and 1.

#### Pixel-based clustering.

The first dimensionality reduction step in our analysis pipeline using spatiomic (33) involved configuring a self-organizing map (SOM) with a grid of 75x75 nodes, trained for 100 iterations using default parameters. The resulting SOM nodes were used to construct a k-nearest neighbor (k-NN) graph with 100 neighbors per node. We then used the Leiden clustering algorithm with a resolution of 3.2 and 500 iterations to effectively cluster the data. Cluster 19 was automatically labeled as background as the maximum normalized pixel intensity values across all markers at the location of this cluster did not exceed 0.15.

#### Downstream analysis.

To extract biologically meaningful patterns from the clusters, we analyzed the output of the *cluster_contributors* function in the spatiomic library. Based on strong similarity in marker intensity patterns, we manually merged clusters 8, 9, and 15 into cluster 4. The main marker contributors from the clusters were then used to manually annotate the cluster IDs in the spatial proximity plots. This was done using the *vicinity_graph* function, which visualizes the spatial adjacency of 2 cluster IDs in the respective clustered result images. The thickness of the edges represents the fraction of the neighborhood pixels occupied by the cluster ID to which the arrow points. In addition, the color of the edges indicates changes concerning the respective reference group, where darker red hues indicate an increase in neighborhood occupation of the cluster, and blue values indicate a decrease. We have excluded isolated and background nodes from this plot to focus on the relevant information. The code for the spatial proteomics analysis will be made available at https://doi.org/10.5281/zenodo.16268042 upon acceptance or reviewer request.

### Immunoblotting

For Western blot analysis, snap-frozen kidney samples were lysed for 30 minutes in 0.5–1 mL of RIPA buffer with the Minilys personal homogenizer (P000673-MLYS0-A, Bertin Technologies). Subsequently, samples were centrifuged at 3,500*g* for 15 minutes, the supernatant was transferred to a new tube for the adjustment of protein concentration. Protein content was determined by a BCA assay (23227, Thermo Fisher Scientific) with a Tecan sunrise scanner (30111999, Tecan Trading AG). Before protein electrophoresis, the samples were diluted with 2x Laemmli buffer (1:1) and cooked at 95°C for 5 minutes. For each lane, 50 μg of protein were loaded. The sample proteins and a protein ladder in one lane were separated in precast gels of 4-20% (4561096EDU, BioRad) for 20 minutes at 80 V and 1 h at 120 V. Immunoblotting was performed with Trans-Blot Turbo Mini 0.2 μm PVDF transfer packs (704156, BioRad) for 7 minutes at 25 V, 1.3 A, using a Trans-Blot Turbo Transfer System (1704150, BioRad). Blocking was performed with a 5% bovine-serum-albumin (BSA, A9430, Sigma-Aldrich) PBST solution (0.1% Tween) for 1 h. The membranes were incubated with a primary antibody solution at 4°C overnight. Incubation with horseradish peroxidase-conjugated secondary antibodies was performed at room temperature for 1 h. Imaging was performed after incubation of the membranes with ECL Western Blot Substrate (32209, Thermo Scientific) with a chemiluminescence detector (Amersham Imager 600). Analysis was performed with ImageJ (2.3.0/1.53 f [64-bit]; NIH). The following primary antibodies have been used (all Cell Signaling unless specified): pP44/42-ERK 1/2 (No. 4370), P44/42-ERK ½ (4695), pS51-eIF2α (9721), total eIF2α (2103S), beta-actin (Sigma-Aldrich, A5441). Secondary antibodies used have been: Rabbit (Thermo Scientific, No. A11037), Mouse (Invitrogen, PA1-74421).

### Flow cytometry

For flow cytometry, tissue was minced and incubated in collagenase — 0.5 mg collagenase D (Millipore Sigma, 11088858001) in 1 mL NaCl, 30 mins, at 37°C — and passed through a 100 μm and a 40 μm strainer. Red cell lysis was done with Red cell lysis buffer (Sigma R7757). Live/dead staining (AlexaFluor 750NHS, Thermo Scientific, A20011) was added in 1:1,000 dilution (10 min, room temperature), and staining was detected with the APC-Cy7 laser at the Symphony A3 flow cytometer (BD Bioscience). Evaluation of the data was done with FlowJo (BD Bioscience).

### snRNA-Seq

#### Single nucleus dissociation.

A snap-frozen kidney tissue sample from pig, mouse, or rat was thawed on ice, and 3–4 mm of the core was chopped with a razor blade in a petri dish on ice and homogenized using a Dounce homogenizer (D8938-1 SET, Sigma-Aldrich) in 200 μL ice-cold lysis solution and incubated on ice for 20-30 min with additional 3.8 mL of ice-cold lysis solution. Lysis solution was prepared with Nuclei PURE lysis buffer (NUC-201, Sigma-Aldrich), 1 mM dithiothreitol (D9779, Sigma-Aldrich), and 0.1 % Triton X-100 (NUC-201, Sigma-Aldrich) according to manufacturer protocol, and an RNase inhibitor mix (0.04 U/μL SUPERaseIN RNase Inhibitor [AM 2696, Thermo Fisher]; 0.04 U/μL RNAsin Plus RNase Inhibitor [N2615, Promega]) was added. The single nuclei suspension was filtered through a 30 μm strainer (04-004-2326, Sysmex) and centrifuged at 500*g* for 5 min at 4°C. The pellet obtained from dissociated rat kidney tissue was resuspended in 2% BSA in DPBS with 0.04 U/μL SUPERaseIN RNase Inhibitor and 0.04 U/μL RNAsin Plus RNase Inhibitor and nuclei were counted. The pellet obtained from dissociated mouse and pig kidney tissue was resuspended and incubated for 2 min in 1 mL RBC lysis buffer hybri-max (R7757-100ml, Sigma Aldrich), filtered through a 5 μm strainer (04-004-2323, Sysmex), and washed with 4 mL of ice-cold 0.01% BSA (AM2616, Thermo Fisher) in DPBS (59331C; Sigma) with 0.04 U/μL SUPERaseIN RNase Inhibitor and 0.04 U/μL RNAsin Plus RNase Inhibitor at 500*g* for 5 min at 4°C. The pellet was resuspended in 1% BSA (mouse and pig) in DPBS with 0.04 U/μL SUPERaseIN RNase Inhibitor and 0.04 U/μL RNAsin Plus RNase Inhibitor, and nuclei were counted.

#### Nuclei loading to the Chromium 10X platform.

Nuclei number was counted and samples were diluted before loading to the Chromium 10X device to capture ~ 10,000 nuclei. The nuclei were separated into Gel Bead Emulsion droplets and libraries were prepared with the Chromium NEXT GEM Single Cell 3’ Reagent kits v3.1 according to manufacturer’s protocol. The libraries were sequenced on an Illumina Novaseq6000 platform as symmetrically paired end runs (150 bases) with 200 million raw sequencing reads per sample.

#### Dataset processing.

Rat and pig reference genomes were generated using Cell Ranger mkref using ENSEMBL mRatBN7.2 (GCA_015227675.2), Sscrofa11.1 (GCA_000003025.6), and for mice, mm10-2020-A was used. For each of the species, the 6 samples (3 perfused, 3 unperfused) were aligned using 10x Genomics Cell Ranger (v.5.0.1) count resulting in 95312, 60035, and 30714 cells for mouse, pig, and rat data, respectively. SoupX (v1.6.1) was used for ambient RNA removal (44). Cells were filtered using a maximum of 5,000 genes per cell and a minimum of 500 genes per cell. Cells with more than 7% mitochondrial gene expression were removed. Scrublet (v0.2.2) was used for doublet removal (45). After these filtering steps, 72,213 (mouse), 52,713 (pig), and 27,152 (rat) cells were further processed. The data were normalized using Seurat’s (v4.0.4) NormalizeData function, including normalization to a total count of 1e4 and subsequent log1p transformation. Highly variable feature selection (2,000 features) was performed using Seurat (v4.0.4) with default settings (46).

For each species, the samples were integrated using Seurat Canonical correlation analysis. Uniform Manifold Approximation and Projection (UMAP) representations were computed on the first 20 principal components. Cell clustering was performed using the Louvain method with a resolution of 0.3.

#### Differential gene expression analysis.

Cluster marker gene detection was performed using Wilcoxon Rank Sum tests with multiple testing corrections (Bonferroni). For the detection of cluster-specific marker genes, only genes that were expressed in more than 25% of the cells and had a log_2_ fold change > 0.25 were considered. For the comparison of perfused vs. unperfused cells, each cell type was considered separately and no cutoffs were applied.

#### Pathway enrichment.

Pathway enrichment was performed using Ingenuity Pathway Analysis (IPA 01-22-01, Qiagen). Positive and negative *z*-score values indicate the activation or inhibition of metabolomic pathways, respectively.

#### Trajectory inference and pseudo-time estimation.

Trajectory inference and pseudo-time estimation were performed on the subset of mouse proximal tubule cells using slingshot (v2.2.1) with default settings (47) using the UMAP representation. No start or end clusters were selected.

#### Hallmark signatures.

To access the cellular signatures for stress response, hallmarks were calculated using Seurat’s AddModuleScore method and the hallmark gene sets APOPTOSIS (MM3869), HYPOXIA (MM3861), OXIDATIVE_PHOSPHORYLATION (MM3893), PEROXISOME (MM3904), and UNFOLDED_PROTEIN_RESPONSE (MM3883) from MSigDB (48). Additionally, a cytokine gene set consisting of *Tnfsf12, Il10, Wnt3a, Nampt, Csf1, Il33, Il3, Prl, Ifng, Il15,* and *Il4* was used to access the cytokine production. For comparison, the 2-tailed Student’s *t* test was used with multiple testing corrections using the Bonferroni method.

#### Compound scores used in Figure 3G.

ToppFun suite was used to generate ranking for scoring percentages of genes in annotation for GO:terms (GO cellular compound, GO molecular function, GO biological process), pathways, and gene family (49). The top hit (e.g., the pathway/GO:term with the highest percentage of genes in annotation) was used.

### Bulk RNA-Seq

#### Library preparation and sequencing.

RNA (2 ng) was used as input material. Libraries were prepared with a SMART-Seq Stranded Kit according to the manual (Takara Bio USA, Mountain View, CA, USA). Library samples were quantified using Quant-iT PicoGreen dsDNA Reagent (Invitrogen; Thermo Fisher Scientific, Waltham, MA, USA) on a ClarioStar microplate reader according to the manufacturer’s instructions (BMG LABTECH, Ortenberg, Germany). The quality, including fragment size, of the cDNA was assessed on an Agilent Technologies Bioanalyzer 2100 using an Agilent DNA 1000 kit according to the manufacturer’s instructions (Agilent Technologies, Palo Alto, CA, USA). Pooled samples were quantified with a Qubit 1X dsDNA HS Assay Kit on a Qubit fluorometer (Thermo Fisher Scientific, Waltham, MA, USA). Single-read sequencing was performed on a NovaSeq 6000 device using an S2 Reagent kit (100 cycles) according to the manufacturer’s instructions (Illumina Inc., CA, USA).

#### Bulk RNA-Seq data analysis.

The quality of the bulk RNA-Seq reads was assessed using FastQC (v0.11.5) (50), and the reads were aligned to the mouse reference genome (mm10) with Bowtie2 (v2.3.3.1) (51) using RSEM (v1.3.0) (52) with the default parameters. The function rsem-calculate-expression was used to align the reads and quantify the gene and isoform abundance. The output of rsem-calculate-expression separately gives the read count and transcripts per million (TPM) value for each gene and isoform. Differential expression analysis was carried out using gene read counts with the DESeq2 package (v1.22.2) (53) to produce log2FC values and corresponding *P* values and adjusted *P* values.

#### qPCR.

Total RNA was extracted with QIAzol Lysis Reagent (79306, Qiagen), and the isolation was performed using miRNeasy Plus Universal Mini Kit (217084, Qiagen). The RNase-Free DNase Set (79254, Qiagen) was used for DNA digestion. For reverse transcription, Protoscript II First Strand cDNA Synthesis Kit (E6560L, New England BioLabs) was used. qPCR was performed on a QuantStudio 3 System (A28566, Thermo Fisher Scientific) using 0.5 μg of cDNA, gene-specific TaqMan primers (see list below), and TaqMan Fast Universal PCR Master Mix (43-660-73, Thermo Fisher Scientific). Glyceraldehyde-3-phosphate dehydrogenase (GAPDH, Thermo Fisher Scientific) was used for normalization. The qPCR data were analyzed with the double delta CT method. The following mouse primers have been used (all Thermo Fisher Scientific): GAPD (GAPDH) (Article number 4352932E, ID Mm99999915_g1), Activating transcription factor 4 (ATF4) (Article number 4331182, ID Mm00515324_m1), DNA damage-inducible transcript 3 (CHOP) (Article number 4331182, ID Mm00492097_m1), Receptor-interacting serine-threonine kinase 3 (RIPK3) (Article number 4331182, ID Mm00444947_m1), and Isocitrate dehydrogenase 1 (IDH1) (Article number 4448892, Mm00516029_m1).

### Metabolomics

Mouse, rat, and pig kidney tissues were lysed in MeOH:H_2_O:CHCl_3_ (1:1:1 *v:v:v,* containing internal standards) at equal tissue to solvent concentrations using a Precellys24 homogenizer as previously described (54). After centrifugation at 15,000*g* and 4°C for 10 min, CHCl_3_ was evaporated to gain a lipid pellet while the MeOH:H_2_O phase was aliquoted for gas chromatography/mass spectrometry as well as for liquid chromatography/targeted mass spectrometry. In each analysis, samples were analyzed in randomized order and pooled quality control samples were regularly injected between the samples to monitor analytical performance. Details of the LC/MS analysis can be found in Lagies et al. (55). Features of targeted analyses were identified by retention time and MRM-transitions obtained from authentic standards. Features of GC/MS analysis were identified by matching spectral fingerprints and retention index information available from 3 different commercially available databases in addition to an in-house database derived from authentic standards. Intensities were normalized to internal standards and the sum of all peaks. For mouse samples, a batch correction using auto-scaling was performed (56).

#### Organelle prediction.

Prediction of organelles involved through analysis of the regulated enriched metabolites was carried out using the metabolite set enrichment analysis feature of MetaboAnalyst 5.0 (57), which returns results for predicted organs and organelles based on the altered metabolites. Results were interpreted based on the number of compound hits and *P* values, organs were omitted and only organelles considered.

#### Pathway enrichment.

Metabolite pathway enrichment was performed using Ingenuity Pathway Analysis (IPA 01-22-01, Qiagen). Positive and negative *z*-score values indicate the activation or inhibition of metabolomic pathways, respectively.

### Statistics

*P* <0.05 was considered to be statistically significant. Two tailed *t* test or 1-way ANOVA were used as specified in the corresponding figures.

### Study approval

#### Mouse.

All procedures were performed under the approval of the University Hospital Hamburg and the Hamburg State Department for Animal Welfare (N002/2020). Experiments were conducted in male C57BL/6 WT mice aged between 3 and 8 months housed under pathogen-free conditions at the animal facility of the University Hospital Hamburg. Mice were maintained at a 12/12 h light/dark cycle and had free access to chow and water *ad libitum*. Animals were age and weight-matched for each experimental series.

#### Rat.

All procedures were performed under the approval of the University Hospital Hamburg and the Hamburg State Department for Animal Welfare (N001/2020). Male Lewis rats of ~250 g body weight were purchased at Charles River Laboratory and were housed under pathogen-free conditions at the animal facility of the University Hospital Hamburg. Rats were maintained at a 12/12 h light/dark cycle and had free access to chow and water ad libitum. Animals were age and weight-matched for each experimental series.

#### Pig.

German landrace pig kidneys were obtained from a local slaughterhouse, where timed slaughter and kidney removal took place. All procedures thereafter took place on organs ex vivo under the approval of the local animal welfare committee of the University Hospital Hamburg (20200518).

#### Human.

The local ethics committee of the University of Rotterdam approved all human tissue protocols (MEC-2022-0290). Human kidney grafts deemed unsuitable for transplantation were sent to the Rotterdam perfusion lab and stored using hypothermic machine perfusion. The study met all criteria of the code of conduct for the responsible use of human tissue that is used in the Netherlands. The study was performed in accordance with the Declaration of Helsinki.

### Data availability

The latest development version of spatiomic can be accessed from GitHub (https://github.com/complextissue/spatiomic). The documentation for spatiomic is available at: https://spatiomic.org and the specific code for the pipeline used in this manuscript will be made available via Zenodo at https://doi.org/10.5281/zenodo.16268042 upon acceptance or reviewer request. Raw and processed snRNA-Seq data is available at GSE218413 and GSE243729. Access to human raw data is limited to the following conditions: Academic research, workplace in EU, signed data transfer agreement with the UKE. All figure data can be accessed and downloaded from a separate Supporting Data Values file.

## Author contributions

JC, F Hausmann, RCM, TBH, and VGP designed the study. JC, SL, SEG, S Christiansen, F Hass, YO, DK, S Hofmann, NS, SB, YF, GA, IE, S Chilla, AKM, and MNW conducted the experiments. JC, F Hass, SL, SEG, NK, YO, DK, MZ, and OK acquired the data. JC, F Hausmann, MZ, TY, ML, FG, MJH, RDB, MK, S Hänzelmann, BK, S Bonn, RCM, TBH, RF, LG, and VGP analyzed the data. JC, RCM, TBH, and VGP wrote the final manuscript. The order of the shared first authors was determined by alphabetical sorting.

## Supplementary Material

Supplemental data

Supporting data values

## Version 1 09/23/2025

Electronic publication

## Version 2 09/24/2025

Patent number added: JC, TBH, and VGP are patent (EP4520171A1) holders for the composition of machine perfusion buffers
