# Supplementary material for: Metabolite-enhanced normothermic machine perfusion improves kidney transplant viability
Source: JCI Insight. 2025 Sep 23;10(18):e190185. doi: 10.1172/jci.insight.190185 (PMC12487851; doi:10.1172/jci.insight.190185)
Supplement: Supplemental data [file jciinsight-10-190185-s282.pdf]

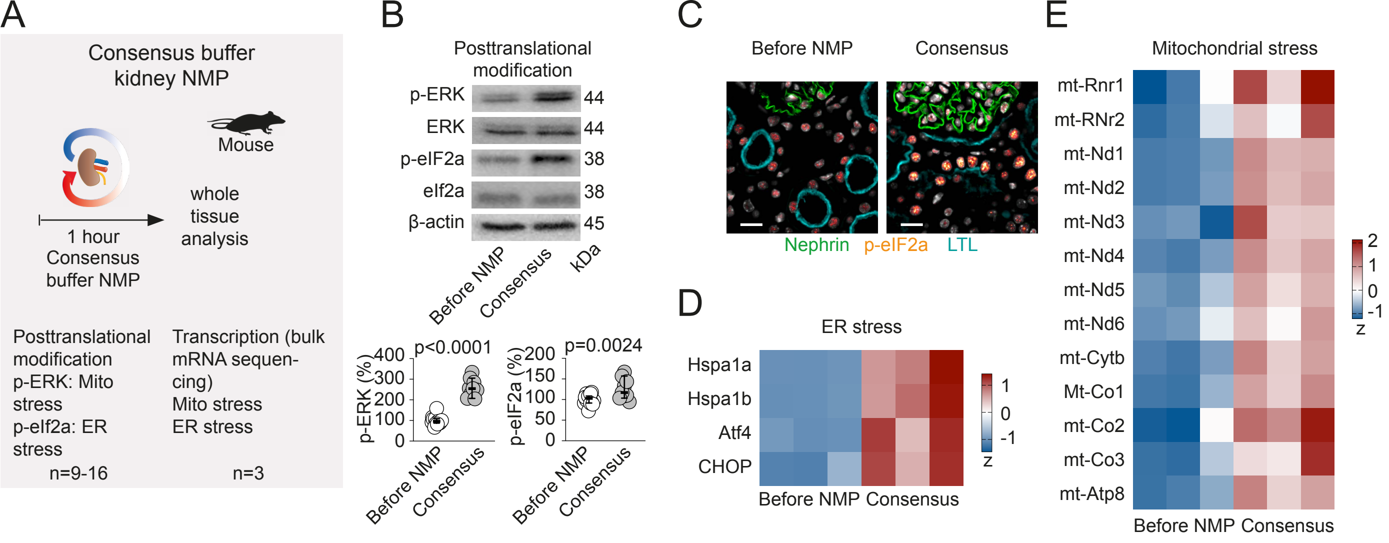
A
Consensus buffer kidney NMP
Mouse
whole tissue analysis
1 hour Consensus buffer NMP
Posttranslational modification
p-ERK: Mito stress
p-eIf2a: ER stress
n=9-16
Transcription (bulk mRNA sequencing)
Mito stress
ER stress
n=3
B
Posttranslational modification
p-ERK 44
ERK 44
p-eIF2a 38
eIf2a 38
β-actin 45
Before NMP
Consensus
kDa
p<0.0001
p-ERK (%)
400
300
200
100
0
p=0.0024
p-eIF2a (%)
200
150
100
50
0
Before NMP
Consensus
C
Before NMP
Consensus
Nephrin
p-eIF2a
LTL
D
ER stress
Hspa1a
Hspa1b
Atf4
CHOP
1
0
-1
z
Before NMP
Consensus
E
Mitochondrial stress
mt-Rnr1
mt-RNr2
mt-Nd1
mt-Nd2
mt-Nd3
mt-Nd4
mt-Nd5
mt-Nd6
mt-Cytb
Mt-Co1
mt-Co2
mt-Co3
mt-Atp8
2
1
0
-1
z
Before NMP
Consensus

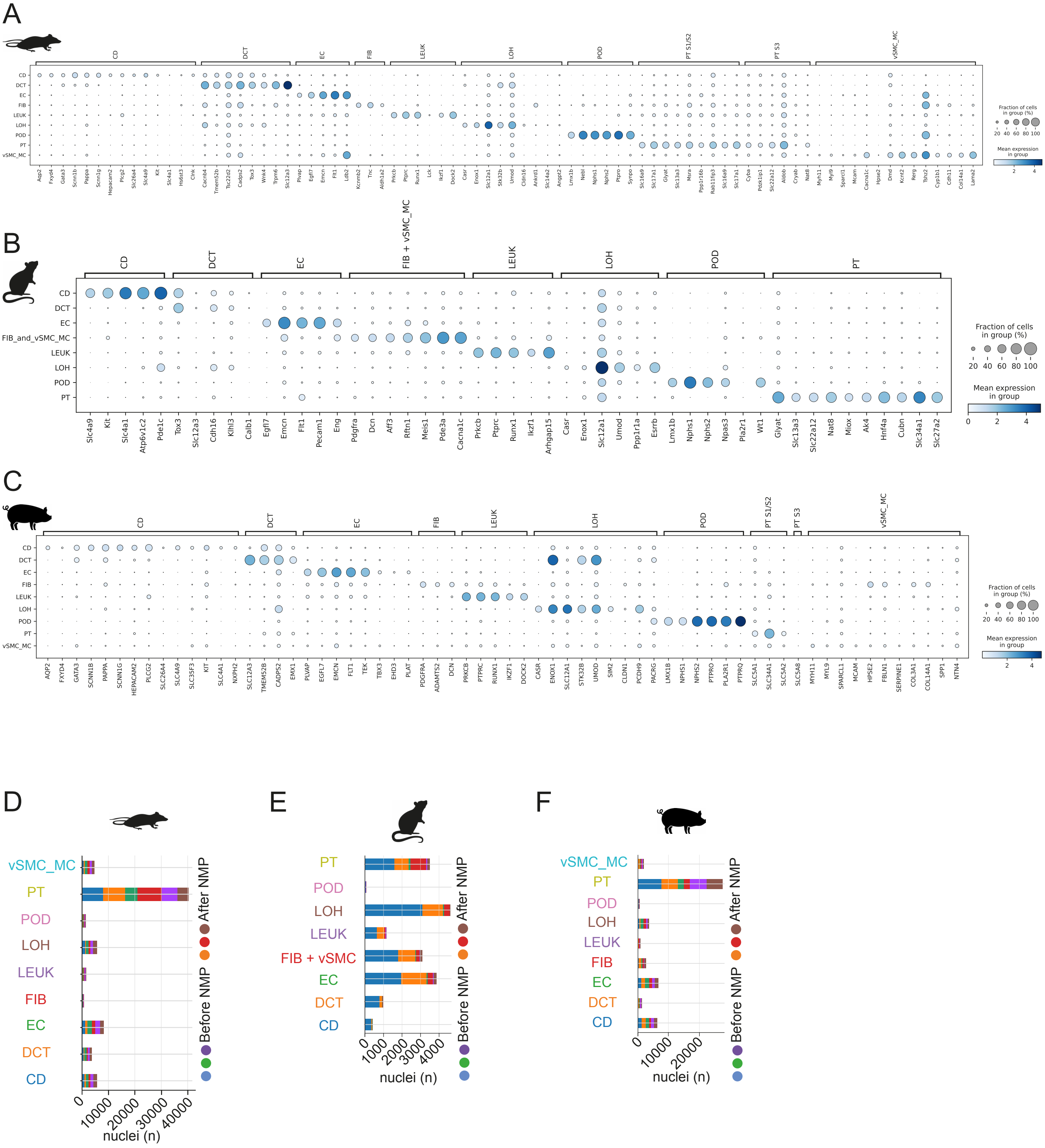

A
CD
DCT
EC
FIB
LEUK
LOH
POD
PT S1/S2
PT S3
vSMC_MC
PT
Fraction of cells in group (%)
20 40 60 80 100
Mean expression in group
2 4
B
CD
DCT
EC
FIB + vSMC_MC
LEUK
LOH
POD
PT
FIB_and_vSMC_MC
Slc4a9
Kit
Slc4a1
Atp6v1c2
Pde1c
Tox3
Slc12a3
Cdh16
Klhl3
Calb1
Egfl7
Emcn
Flt1
Pecam1
Eng
Pdgfra
Dcn
Aff3
Rftn1
Meis1
Pde3a
Cacna1c
Prkcb
Ptprc
Runx1
Ikzf1
Arhgap15
Casr
Enox1
Slc12a1
Umod
Ppp1r1a
Esrrb
Lmx1b
Nphs1
Nphs2
Npas3
Pla2r1
Wt1
Glyat
Slc13a3
Slc22a12
Nat8
Miox
Ak4
Hnf4a
Cubn
Slc34a1
Slc27a2
Fraction of cells in group (%)
20 40 60 80 100
Mean expression in group
0 2 4
C
CD
DCT
EC
FIB
LEUK
LOH
POD
PT S1/S2
PT S3
vSMC_MC
PT
AQP2
FXYD4
GATA3
SCNN1B
PAPPA
SCNN1G
HEPACAM2
PLCG2
SLC26A4
SLC4A9
SLC35F3
KIT
SLC4A1
NXPH2
SLC12A3
TMEM52B
CADPS2
EMX1
PLVAP
EGFL7
EMCN
FLT1
TEK
TBX3
EHD3
PLAT
PDGFRA
ADAMTS2
DCN
PRKCB
PTPRC
RUNX1
IKZF1
DOCK2
CASR
ENOX1
SLC12A1
STK32B
UMOD
SIM2
CLDN1
PCDH9
PACRG
LMX1B
NPHS1
NPHS2
PTPRO
PLA2R1
PTPRQ
SLC5A1
SLC34A1
SLC5A2
SLC5A8
MYH11
MYL9
SPARCL1
MCAM
HPSE2
FBLN1
SERPINE1
COL3A1
COL14A1
SPP1
NTN4
Fraction of cells in group (%)
20 40 60 80 100
Mean expression in group
2 4
D
vSMC_MC
PT
POD
LOH
LEUK
FIB
EC
DCT
CD
0 10000 20000 30000 40000
nuclei (n)
After NMP
Before NMP
E
PT
POD
LOH
LEUK
FIB + vSMC
EC
DCT
CD
0 1000 2000 3000 4000
nuclei (n)
After NMP
Before NMP
F
vSMC_MC
PT
POD
LOH
LEUK
FIB
EC
DCT
CD
0 10000 20000
nuclei (n)
After NMP
Before NMP

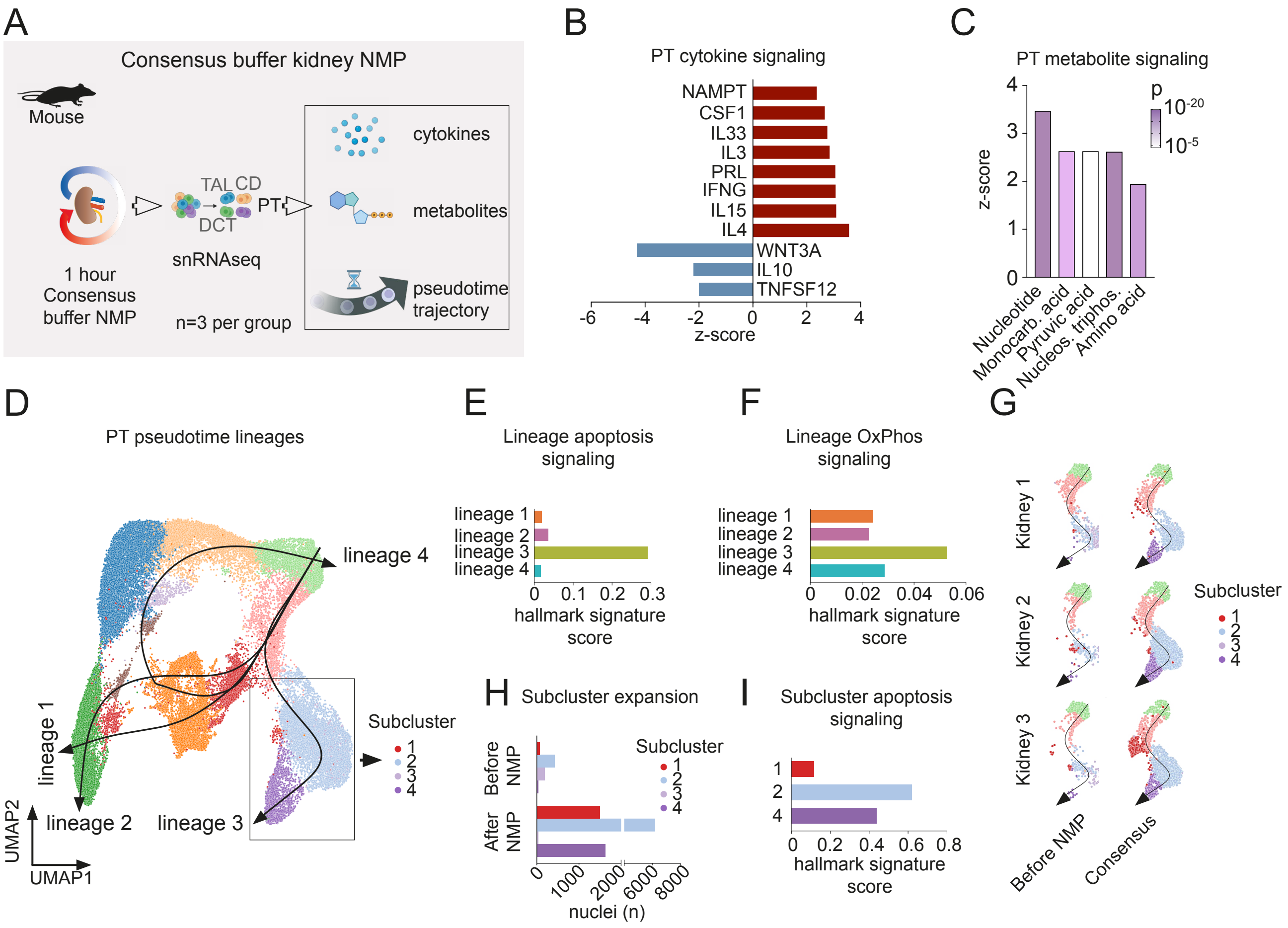
A
Consensus buffer kidney NMP
Mouse
1 hour
Consensus
buffer NMP
TAL CD
DCT
PT
snRNAseq
n=3 per group
cytokines
metabolites
pseudotime
trajectory
B
PT cytokine signaling
NAMPT
CSF1
IL33
IL3
PRL
IFNG
IL15
IL4
WNT3A
IL10
TNFSF12
z-score
C
PT metabolite signaling
z-score
p
10^{-20}
10^{-5}
Nucleotide
Monocarb. acid
Pyruvic acid
Nucleos. triphos.
Amino acid
D
PT pseudotime lineages
lineage 1
lineage 2
lineage 3
lineage 4
Subcluster
UMAP1
UMAP2
E
Lineage apoptosis
signaling
hallmark signature
score
F
Lineage OxPhos
signaling
hallmark signature
score
G
Kidney 1
Kidney 2
Kidney 3
Subcluster
Before NMP
Consensus
H
Subcluster expansion
Before
NMP
After
NMP
nuclei (n)
I
Subcluster apoptosis
signaling
hallmark signature
score

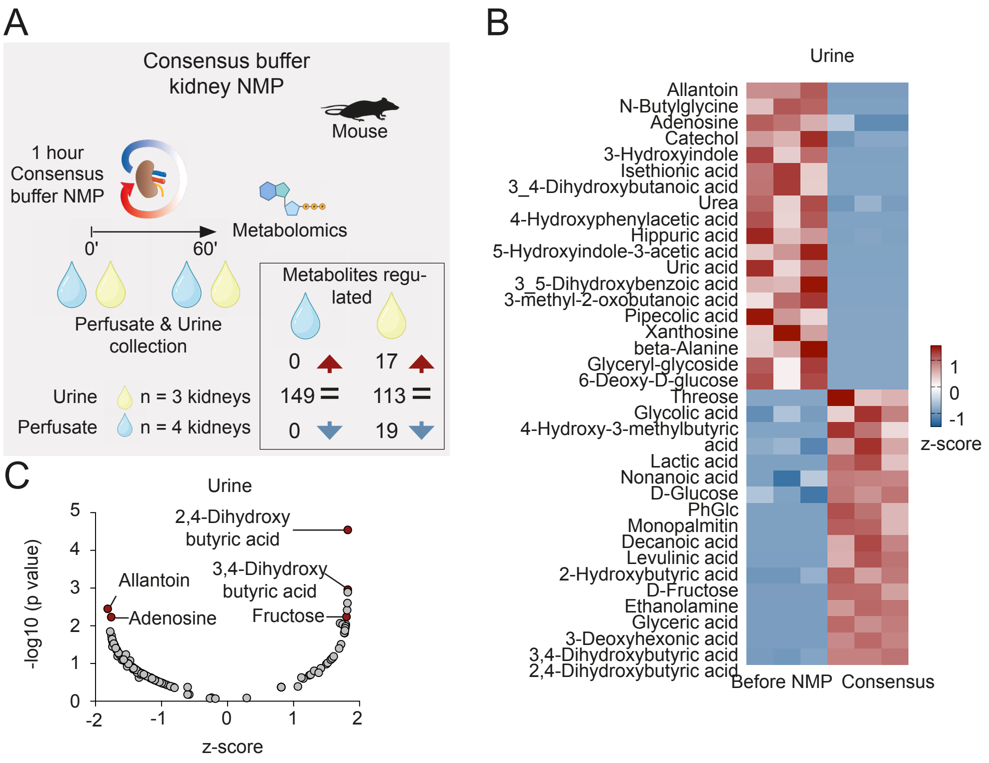
A
Consensus buffer kidney NMP
Mouse
1 hour Consensus buffer NMP
Metabolomics
0'
60'
Perfusate & Urine collection
Metabolites regulated
0
149
0
17
113
19
Urine n = 3 kidneys
Perfusate n = 4 kidneys
B
Urine
Allantoin
N-Butylglycine
Adenosine
Catechol
3-Hydroxyindole
Isethionic acid
3_4-Dihydroxybutanoic acid
Urea
4-Hydroxyphenylacetic acid
Hippuric acid
5-Hydroxyindole-3-acetic acid
Uric acid
3_5-Dihydroxybenzoic acid
3-methyl-2-oxobutanoic acid
Pipecolic acid
Xanthosine
beta-Alanine
Glyceryl-glycoside
6-Deoxy-D-glucose
Threose
Glycolic acid
4-Hydroxy-3-methylbutyric acid
Lactic acid
Nonanoic acid
D-Glucose
PhGlc
Monopalmitin
Decanoic acid
Levulinic acid
2-Hydroxybutyric acid
D-Fructose
Ethanolamine
Glyceric acid
3-Deoxyhexonic acid
3,4-Dihydroxybutyric acid
2,4-Dihydroxybutyric acid
Before NMP
Consensus
1
0
-1
z-score
C
Urine
-log10 (p value)
2,4-Dihydroxy butyric acid
3,4-Dihydroxy butyric acid
Allantoin
Adenosine
Fructose
z-score
5
4
3
2
1
0
-2
-1
0
1
2

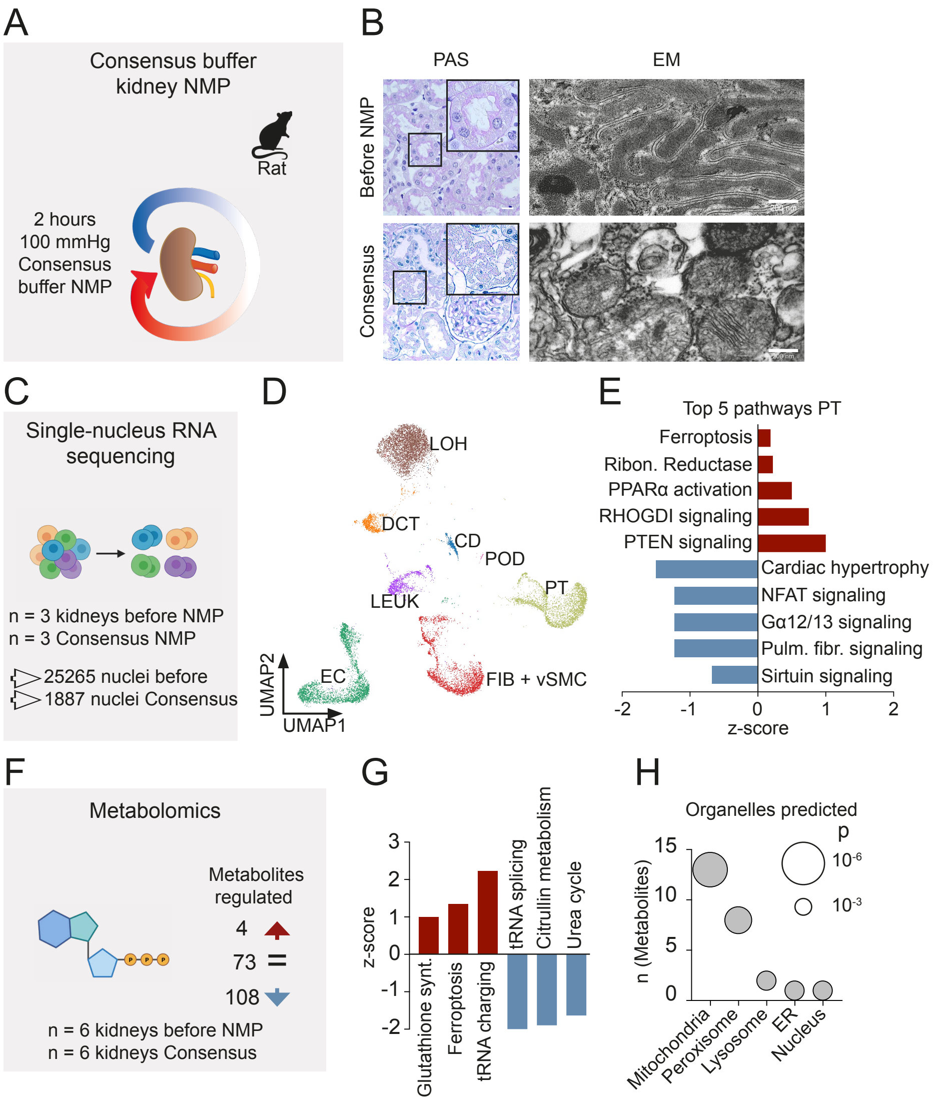
A
Consensus buffer kidney NMP
Rat
2 hours
100 mmHg
Consensus buffer NMP
B
PAS
EM
Before NMP
Consensus
C
Single-nucleus RNA sequencing
n = 3 kidneys before NMP
n = 3 Consensus NMP
25265 nuclei before
1887 nuclei Consensus
D
LOH
DCT
CD
POD
PT
LEUK
EC
FIB + vSMC
UMAP2
UMAP1
E
Top 5 pathways PT
Ferroptosis
Ribon. Reductase
PPARα activation
RHOGDI signaling
PTEN signaling
Cardiac hypertrophy
NFAT signaling
Gα12/13 signaling
Pulm. fibr. signaling
Sirtuin signaling
-2
-1
0
1
2
z-score
F
Metabolomics
Metabolites regulated
4
73
108
n = 6 kidneys before NMP
n = 6 kidneys Consensus
G
z-score
3
2
1
0
-1
-2
Glutathione synt.
Ferroptosis
tRNA charging
tRNA splicing
Citrullin metabolism
Urea cycle
H
Organelles predicted
p
10-6
10-3
n (Metabolites)
15
10
5
0
Mitochondria
Peroxisome
Lysosome
ER
Nucleus

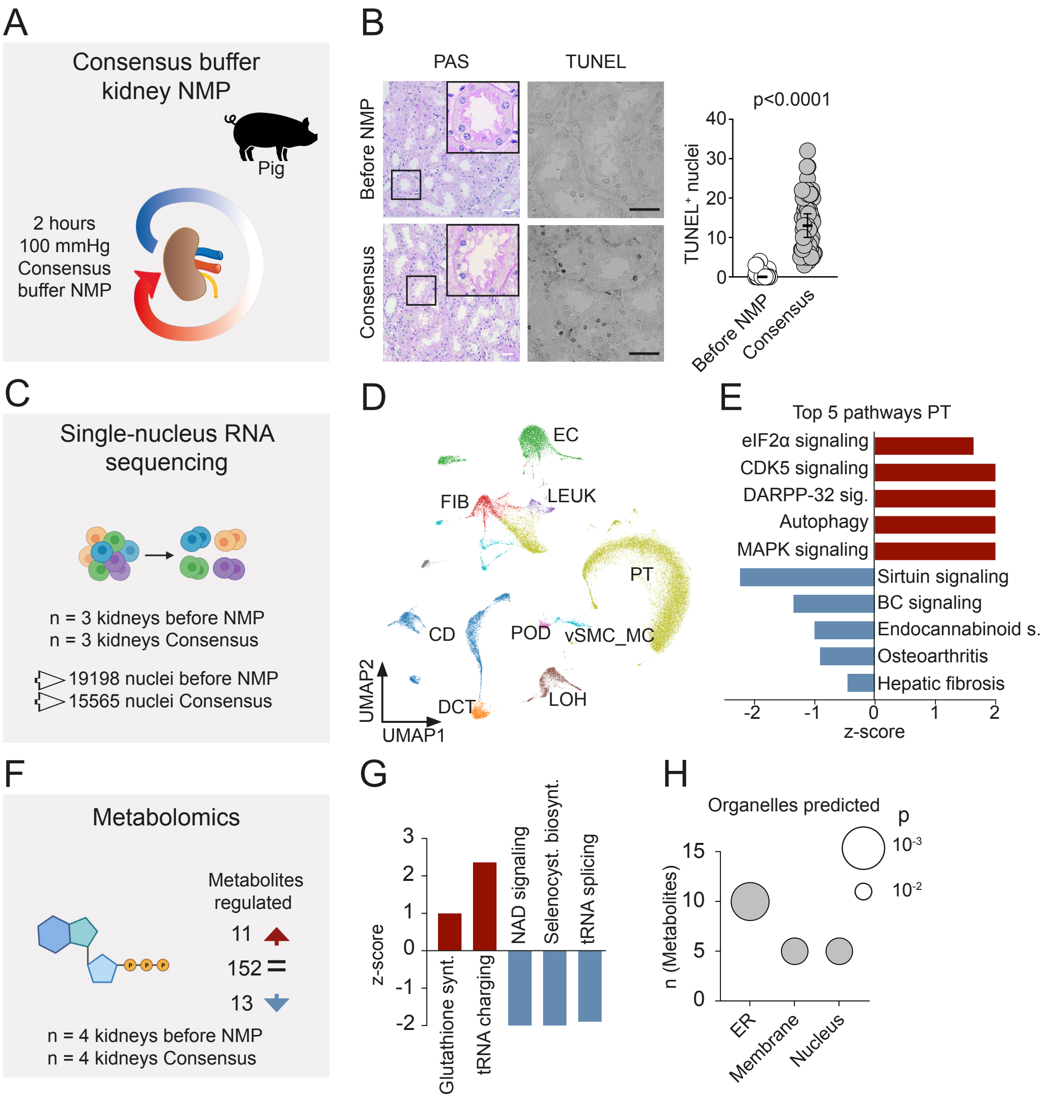

A
Consensus buffer kidney NMP
Pig
2 hours
100 mmHg
Consensus buffer NMP
B
PAS
TUNEL
Before NMP
Consensus
p<0.0001
TUNEL⁺ nuclei
Before NMP
Consensus
C
Single-nucleus RNA sequencing
n = 3 kidneys before NMP
n = 3 kidneys Consensus
19198 nuclei before NMP
15565 nuclei Consensus
D
EC
FIB
LEUK
PT
CD
POD
vSMC_MC
DCT
LOH
UMAP1
UMAP2
E
Top 5 pathways PT
eIF2α signaling
CDK5 signaling
DARPP-32 sig.
Autophagy
MAPK signaling
Sirtuin signaling
BC signaling
Endocannabinoid s.
Osteoarthritis
Hepatic fibrosis
z-score
F
Metabolomics
Metabolites regulated
11
152
13
n = 4 kidneys before NMP
n = 4 kidneys Consensus
G
z-score
Glutathione synt.
tRNA charging
NAD signaling
Selenocyst. biosynt.
tRNA splicing
H
Organelles predicted
p
10-3
10-2
n (Metabolites)
ER
Membrane
Nucleus

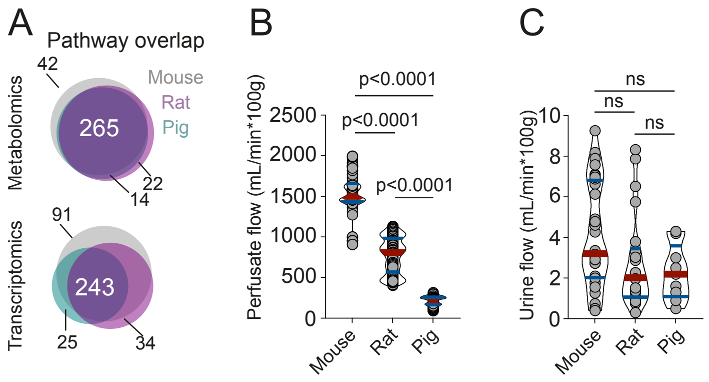
A
Pathway overlap
Metabolomics
Transcriptomics
Mouse
Rat
Pig
42
265
22
14
91
243
25
34
B
Perfusate flow (mL/min*100g)
p<0.0001
p<0.0001
p<0.0001
2500
2000
1500
1000
500
0
Mouse
Rat
Pig
C
Urine flow (mL/min*100g)
ns
ns
ns
10
8
6
4
2
0
Mouse
Rat
Pig

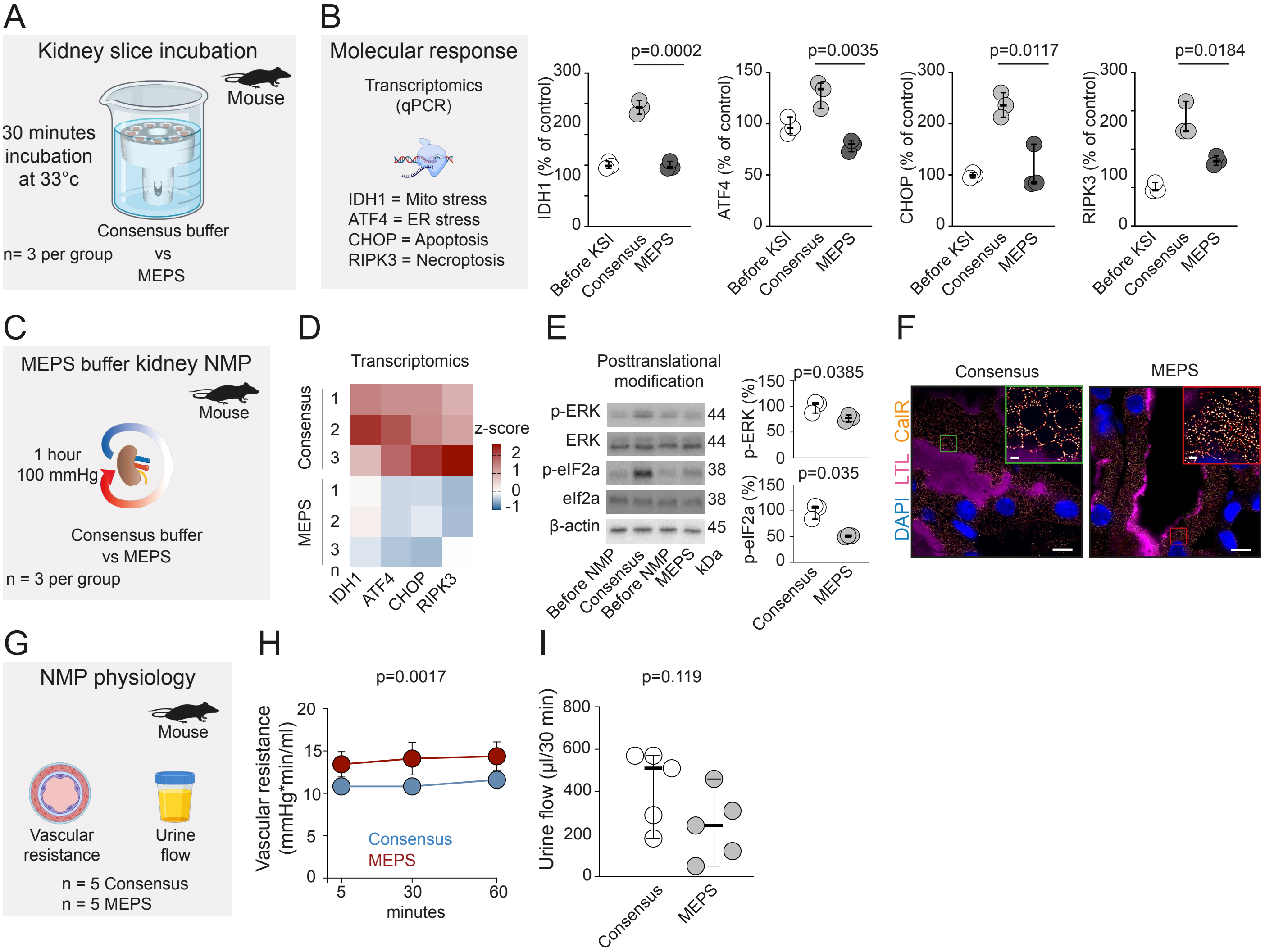
A
Kidney slice incubation
Mouse
30 minutes incubation at 33°c
Consensus buffer vs MEPS
n= 3 per group
B
Molecular response
Transcriptomics (qPCR)
IDH1 = Mito stress
ATF4 = ER stress
CHOP = Apoptosis
RIPK3 = Necroptosis
p=0.0002
p=0.0035
p=0.0117
p=0.0184
IDH1 (% of control)
ATF4 (% of control)
CHOP (% of control)
RIPK3 (% of control)
Before KSI
Consensus
MEPS
C
MEPS buffer kidney NMP
Mouse
1 hour
100 mmHg
Consensus buffer vs MEPS
n = 3 per group
D
Transcriptomics
Consensus
MEPS
z-score
IDH1
ATF4
CHOP
RIPK3
E
Posttranslational modification
p-ERK
ERK
p-eIF2a
eIf2a
β-actin
kDa
Before NMP
Consensus
Before NMP
MEPS
p=0.0385
p=0.035
p-ERK (%)
p-eIF2a (%)
F
Consensus
MEPS
DAPI LTL CalR
G
NMP physiology
Mouse
Vascular resistance
Urine flow
n = 5 Consensus
n = 5 MEPS
H
p=0.0017
Vascular resistance (mmHg*min/ml)
Consensus
MEPS
minutes
I
p=0.119
Urine flow (µl/30 min)
Consensus
MEPS

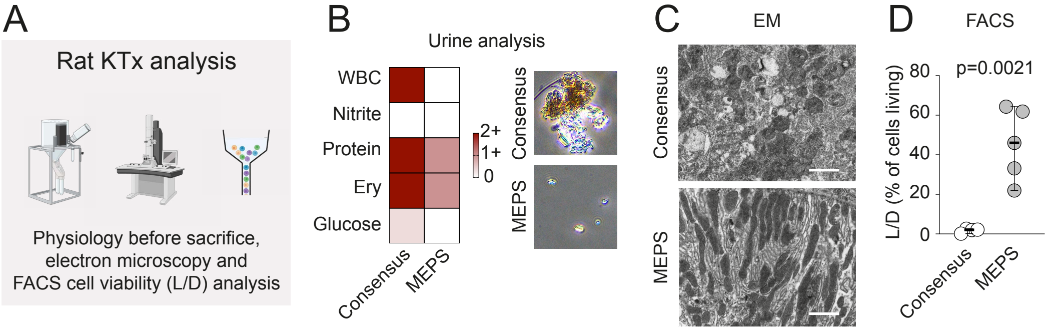

E

Cause of death of individual animals

Consensus

Animal 1	anuric; found dead 12h after KTx
Animal 2	pain/akinesia; sacrificed 48h after KTx
Animal 3	no food/water uptake; sacrificed 48h after KTx
Animal 4	anuric; found dead 24h after KTx
Animal 5	anuric; dead 24h after KTx, venous thrombosis
Animal 6	postren. sten. + renal ne-cros.; dead 48h post Tx

MEPS

Animal 1	postrenal failure d9
Animal 2	sacrificed at end
Animal 3	sacrificed at end
Animal 4	postrenal failure d11
Animal 5	sacrificed at end

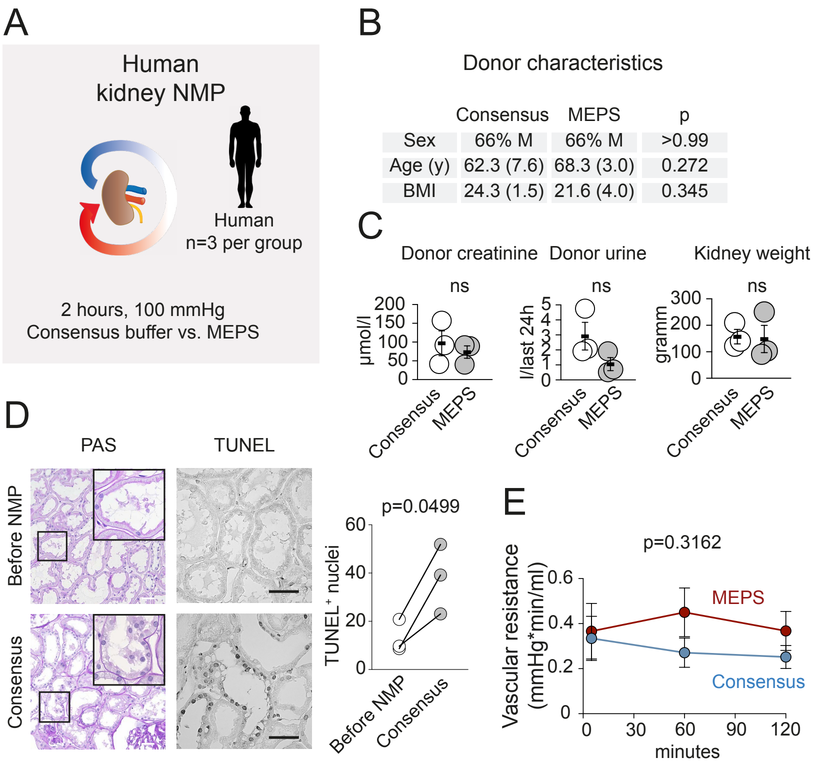
A
Human
kidney NMP
Human
n=3 per group
2 hours, 100 mmHg
Consensus buffer vs. MEPS
B
Donor characteristics
Consensus MEPS p
Sex 66% M 66% M >0.99
Age (y) 62.3 (7.6) 68.3 (3.0) 0.272
BMI 24.3 (1.5) 21.6 (4.0) 0.345
C
Donor creatinine
Donor urine
Kidney weight
ns
µmol/l
l/last 24h
gramm
Consensus
MEPS
D
PAS
TUNEL
Before NMP
Consensus
p=0.0499
TUNEL + nuclei
Before NMP
Consensus
E
p=0.3162
Vascular resistance (mmHg*min/ml)
MEPS
Consensus
minutes
